# Multiparametric Optimization of Fabrication of Electrospun PVA Nanofibers for Utilization as Wound Dressing Mats

**DOI:** 10.3390/polym18162018

**Published:** 2026-08-20

**Authors:** Tshepang Mqatywa, Mehrab Mahdian, Paula Ossowicz-Rupniewska, Karolina Zyburtowicz-Ćwiartka, Karolina Bilska, Anna Nowak, Tímea Beskid, Mária Laki, András József Laki, János Juhász, Tamás Pardy, Franciska Erdő

**Affiliations:** 1 Faculty of Information Technology and Bionics, Pázmány Péter Catholic University, Práter utca 50A, H-1083 Budapest, Hungary; mqatywatshepi98@gmail.com (T.M.);; 2 Thomas Johann Seebeck Department of Electronics, Tallinn University of Technology, 19086 Tallinn, Estonia; 3 Department of Chemical Organic Technology and Polymeric Materials, Faculty of Chemical Technology and Engineering, West Pomeranian University of Technology in Szczecin, Piastów Ave. 42, 71-065 Szczecin, Poland; 4 Faculty of Health Sciences, Pomeranian Medical University in Szczecin, 70-204 Szczecin, Poland; 5 Institute of Medical Microbiology, Semmelweis University, H-1089 Budapest, Hungary; 6 Department of Chemistry and Biotechnology, Tallinn University of Technology, 19086 Tallinn, Estonia

**Keywords:** PVA polymer, wound healing, nanofibers, electrospinning, wound dressings, scanning electron microscopy, TGA, manufacturing parameters, DSC, swelling

## Abstract

Electrospun poly (vinyl alcohol) (PVA) nanofibers are promising materials for wound dressings because of their high porosity, large surface area, and structural similarity to the extracellular matrix. In this study, a Box–Behnken design was used to optimize the effects of applied voltage, flow rate, spinning distance, and needle gauge on the fabrication of electrospun PVA nanofiber mats using an 8% (*w*/*v*) PVA precursor solution. Fiber morphology, diameter, porosity, thickness, swelling capacity, and thermal properties were evaluated. Stable electrospinning conditions produced uniform, bead-free nanofibers with mean diameters ranging from 198.9 ± 3.6 to 228.7 ± 1.5 nm. Needle gauge and flow rate were identified as the most influential parameters affecting fiber diameter, while voltage and spinning distance showed interaction-dependent effects. Thinner fibers generated using finer needles resulted in higher porosity and enhanced swelling behavior, whereas larger needle diameters produced thicker and denser mats. Thermal analysis demonstrated good thermal stability, with degradation onset temperatures ranging from approximately 225 to 280 °C, and melting transitions consistently occurring at 190–193 °C. The results establish clear process–structure–property relationships and demonstrate that multiparametric optimization enables reproducible fabrication of PVA nanofibers with tunable characteristics relevant for wound-dressing applications.

## 1. Introduction

Skin is the largest organ of the human body and serves as a critical barrier against mechanical injury, microbial invasion, dehydration, and environmental stress. Impaired wound healing can result in chronic wounds, infection, prolonged inflammation, and substantial healthcare costs. Therefore, the development of advanced wound dressings capable of maintaining an optimal healing microenvironment remains a major challenge in biomedical materials research [1,2].

Modern wound dressings are expected not only to protect the wound physically but also to regulate moisture, facilitate oxygen exchange, absorb exudate, and promote tissue regeneration [3,4]. Among the emerging technologies developed to address these requirements, electrospun nanofiber mats have attracted considerable attention because of their close structural resemblance to the extracellular matrix (ECM), high porosity, interconnected pore structure, and large surface-area-to-volume ratio [5,6].

Electrospinning is a versatile fiber fabrication technique that employs electrostatic forces to form continuous fibers from polymer solutions or melts. Depending on processing conditions, electrospun fibers can be produced with diameters ranging from several micrometers to tens of nanometers, providing excellent opportunities for tailoring scaffold architecture and functionality [7,8]. Recent studies have further demonstrated the potential of electrospun nanofibers as carriers for antimicrobial agents, anti-inflammatory compounds, antioxidants, and growth factors, thereby enabling localized therapeutic delivery and enhanced wound healing performance [9,10].

Among the polymers used for electrospinning, poly (vinyl alcohol) (PVA) is particularly attractive because of its biocompatibility, biodegradability, non-toxicity, water solubility, and excellent film-forming properties [11,12]. Electrospun PVA nanofibers have shown significant promise in wound healing, tissue engineering, and drug delivery applications owing to their hydrophilic nature and ease of processing [2,13]. However, the performance of PVA nanofiber mats strongly depends on their morphology, including fiber diameter, uniformity, porosity, and thickness, all of which are highly sensitive to electrospinning conditions.

Although electrospinning is relatively simple in principle, successful nanofiber fabrication depends on a complex interaction between solution parameters, process parameters, and environmental factors. Solution characteristics such as polymer concentration, viscosity, conductivity, surface tension, and viscoelastic behavior influence jet stability and fiber formation. Simultaneously, process variables including applied voltage, flow rate, spinning distance, and needle geometry govern electrohydrodynamic stretching, solvent evaporation, and final fiber morphology [5,14]. Recent studies have shown that interactions among these variables often exert a greater influence on fiber formation than individual factors considered independently [15,16]. Polymer concentration is one of the most critical solution parameters governing electrospinning because it directly influences viscosity, molecular-chain entanglement, and jet stability. Based on preliminary screening experiments and previous studies on electrospun PVA systems, an 8% (*w*/*v*) PVA concentration was selected for the present work. Lower concentrations resulted in insufficient chain entanglement and unstable fiber formation, whereas higher concentrations substantially increased solution viscosity and promoted the formation of thicker fibers. The selected concentration provided stable Taylor cone formation and reproducible production of uniform nanofibers within the diameter range desirable for wound-dressing applications.

To address these challenges, Design-of-Experiments (DoE) methodologies have increasingly been applied to electrospinning optimization. Statistical approaches such as Box–Behnken and Taguchi designs enable efficient identification of significant factors while substantially reducing the number of experimental runs required compared with conventional one-factor-at-a-time approaches [17,18]. Moreover, recent developments in surrogate modeling, machine learning, and data-driven process optimization highlight the growing need for comprehensive experimental datasets that capture process–structure–property relationships in electrospinning systems [16,18].

Despite considerable progress in electrospinning optimization relatively few studies have systematically investigated the combined effects of applied voltage, flow rate, spinning distance, and needle gauge on the resulting morphology and functional properties of electrospun PVA wound-dressing materials. Furthermore, the relationship between process stability, Taylor cone behavior, nanofiber morphology, swelling performance, and thermal characteristics remains insufficiently understood.

The novelty of the present study lies in the integration of multiparametric electrospinning optimization with Taylor cone stability assessment and comprehensive characterization of the resulting nanofiber mats. Unlike previous studies that primarily focus on individual electrospinning variables, the present work evaluates the combined effects of applied voltage, flow rate, spinning distance, and needle gauge using two complementary Box–Behnken [19] experimental designs. In addition, relationships between process parameters, fiber morphology, porosity, swelling behavior, thickness, and thermal properties are systematically investigated to provide a broader understanding of process–structure–property interactions in electrospun PVA systems.

Therefore, the objectives of this study were: (i) to systematically evaluate the effects of key electrospinning parameters on PVA nanofiber fabrication; (ii) to identify robust processing conditions that yield uniform and reproducible nanofiber morphologies; (iii) to determine how process-induced morphological variations influence porosity, swelling capacity, thickness, and thermal stability; and (iv) to establish a reproducible framework for optimization of electrospun PVA nanofibers intended for wound-dressing applications.

## 2. Materials and Methods

### 2.1. Materials

Polyvinyl alcohol (PVA) powder with a molecular weight of 130,000 g/mol was obtained from Sigma-Aldrich Chemie GmbH (Steinheim, Germany) and dissolved in distilled water. PVA had a degree of polymerization of approximately 1800 monomer units and 88% degree of hydrolysis. Commercial aluminum foil measuring 15 × 15 cm was used as the substrate for all nanofiber deposition.

### 2.2. Electrospinning Conditions

#### 2.2.1. Precursor Solution Preparation

The polymer solution was prepared from 8 g of PVA powder and 92 g of distilled water. A standard 100 mL GL-45 wide-mouth glass reagent bottle was used to prepare the solution. An IKA RCT basic magnetic stirrer (IKA, Staufen im Breisgau, Germany) gently facilitated the dissolution of PVA at 60 °C and 750 rpm. The solution was then stirred continuously for more than 8 h to ensure complete dissolution and homogenization. The resulting PVA solution had a concentration of 8% (*w*/*v*). Preliminary experiments were performed with other concentrations of PVA and 8% gave the best balance between spinnability and fiber quality (bead-free fibers).

#### 2.2.2. Electrospinning and Experimental Design

A SpinCube electrospinner (Spinsplit, Budapest, Hungary) was employed for all the experiments. The machine was equipped with a flow-controlled pump and a DC high voltage power supply with an output voltage of up to 40 kV. The electrospinner had a horizontal emitter-collector arrangement with single-needle emitters and a 20 × 20 cm stainless-steel flat-plate collector. The distance between the emitter and collector could be adjusted between 5 and 30 cm. All experiments were conducted at a constant PVA concentration of 8% (*w*/*v*), under temperatures of 23–24 °C and relative humidity of 40–70%.

A Box–Behnken experimental design was used to fabricate all nanofibers. Based on the literature, ranges for applied voltage, flow rate, spinning distance, and needle size were selected to produce nanofibers with a small mean fiber diameter, high uniformity (low standard deviation), and adequate porosity.

Two Box–Behnken designs were employed, with the first design (BBD-I) comprising three parameters that included applied voltage, flow rate, and spinning distance, while the second design (BBD-II) involved four parameters with the addition of needle size as a factor. A preliminary set of experiments was conducted to evaluate the stability of the electrospinning process and to further refine the parameter intervals.

BBD-I consisted of 3 factors at 3 levels, while BBD-II had 4 factors at 3 levels. Three points, set at −1 (low), 0 (center point), and 1 (high), were used to define the levels of the experimental design. BBD-I experiments were intended to evaluate the effects of applied voltage, flow rate, and spinning distance on mean fiber diameter, uniformity, and porosity. A constant needle size of 18 G was utilized for all BBD-I experiments. BBD-II also evaluated the influence of the aforementioned factors, but with the addition of varying needle sizes of 16 and 20 G. Additionally, since the 18 G needle was already used to conduct BBD-I experiments, needle sizes were only varied between 16 and 20 G in BBD-II, and their results were analyzed in tandem with the 18 G. BBD-I yielded 15 experimental runs, while BBD-II yielded 16 runs. The formula below was used to calculate the number of experimental runs:

$$N=2k\left(k-1\right)+cp$$

where *N* refers to the number of runs, with *k* annotating the number of factors and *cp* the number of center points. Furthermore, the applied voltage was denoted V, the flow rate Q, the spinning distance D, and the needle size G. Numiqo software (https://numiqo.com) was used to generate combinations of the input parameters according to the Box–Behnken experimental design. Table 1 below shows the parameters of BBD-I and BBD-II at different experimental levels.

#### 2.2.3. Taylor Cone

The SpinCube electrospinner was additionally equipped with an imaging system featuring a monochrome 1.3 MP 10× optical-zoom camera with a 5–50 mm focal length and up to 30 fps. This camera facilitated real-time monitoring of the electrospinning process and image acquisition. During electrospinning, the images of the Taylor cones were captured and labeled according to the predetermined format.

A standardized set of criteria for the qualitative assessment and rating of the Taylor cones was then developed. The objective was to ensure consistent, reproducible labeling of image data to enable later training of a machine learning model for quality control. The Taylor cone criteria used a 4-point qualitative rating scale, focusing on the cone’s shape, jet, and visual cues. Taylor cone shape and jet stability were the primary criteria used for score assignment. Table 2 shows the 1–4 ratings for the Taylor cone criteria.

**Figure 1:**
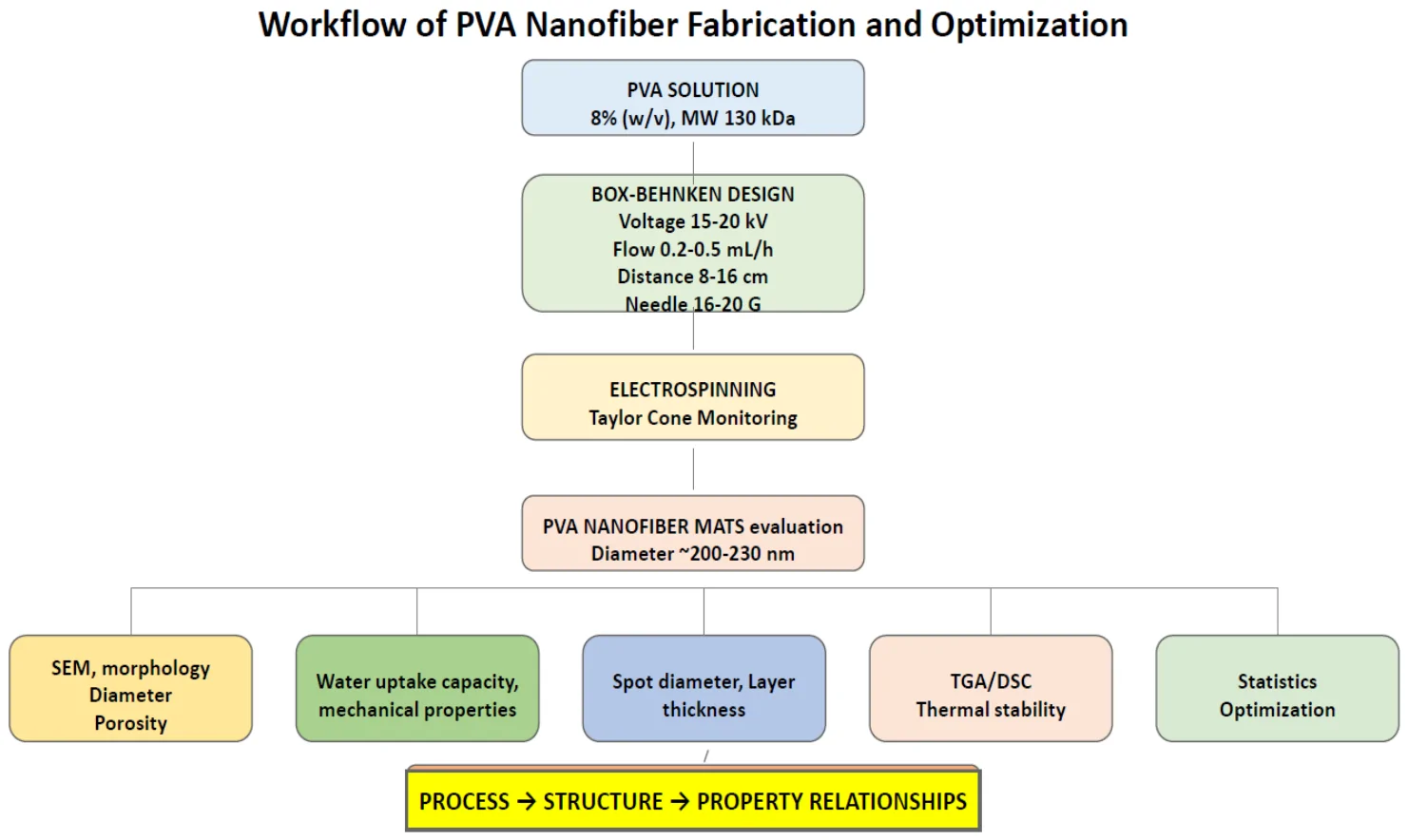
The short workflow of the fabrication and evaluation of PVA-based electrospun nanofiber scaffolds. The consecutive steps of the process are: preparation → processing → characterization → optimization. SEM: scanning electronmicroscopy, TGA: thermogravimetric analysis, DSC: differential scanning calorimetry.

The simplified, PVA-based nanofiber manufacturing and characterization workflow is presented in Figure 1.

### 2.3. Characterization of the End Products

#### 2.3.1. Scanning Electron Microscopy (SEM)

The nanofiber samples were first coated with gold (Au) particles using an MCM-100P sputter coater (SEC Co., Ltd., Suwon-si, Republic of Korea). The fibers were coated under vacuum at low voltage to increase the samples’ conductivity. A preliminary set of experiments was conducted to determine optimal evaluation conditions for nanofiber characterization under SEM. A Hitachi TM4000Plus tabletop SEM (Hitachi, Tokyo, Japan) was used to analyze the samples at a magnification of 3000×, an accelerating voltage of 5 kV, and a working distance of 5.3–6.3 mm. A simultaneous combination of back-scattered electron (BSE) and secondary electron (SE) detectors was used to analyze all samples. The captured SEM micrographs were used to measure the mean fiber diameters, uniformity (standard deviation), and porosity of the samples for each of the three or four electrospinning parameter combinations, using a custom Python image-analysis workflow. All images were acquired at identical magnification (3000×), accelerating voltage (5 kV), and working-distance range. For each nanofiber mat, images were captured from multiple randomly selected regions avoiding edges and visible defects. The Python workflow quantified fiber diameter using thousands of local measurements per image, minimizing operator bias.

#### 2.3.2. Nanofiber Diameters, Uniformity, and Porosity

Fiber morphology was quantified from SEM micrographs using custom Python scripts. Images were converted to grayscale, calibrated using the SEM scale bar (µm per pixel), and cropped to remove annotations. Fibers were segmented by Otsu thresholding followed by morphological filtering, skeletonized to obtain fiber centerlines, and analyzed using a Euclidean distance transform. Local fiber diameters were calculated as twice the distance from the skeleton to the fiber boundary, yielding per-image diameter distributions. Mean fiber diameter and uniformity were reported as the arithmetic mean and standard deviation, respectively. Porosity was estimated as the percentage of the image area occupied by voids after binary segmentation of pores.

#### 2.3.3. Layer Spot Diameter Measurements

The images of the fiber disk depositions at the end of each electrospinning experiment were captured. The diameters of the viable nanofiber disks were then measured using ImageJ software (V 1.54k). Triplicate measurements for each parameter combination in the BBD-I and BBD-II experiments were taken, and the data were reported as mean ± standard deviation (SD).

#### 2.3.4. Nanofiber Layer Thickness

The thickness of the nanofibers dictates the material’s absorption capacity, with thicker mats generally retaining more fluid and exhibiting greater mechanical strength. The breathability and barrier function of the dressings also depend on the thickness of the nanofiber mats, as this thickness influences water vapor transmission, oxygen diffusion, and microbial barrier properties. For instance, fiber dressings that are too thin may leak or tear, while too-thick mats trap moisture and reduce breathability. Thickness measurements were performed on nanofiber mats produced under identical electrospinning deposition times and collector configurations. All samples were measured at three independent locations and reported as mean ± SD. Because deposition time was kept constant for all experimental runs, differences in thickness inherently reflect the influence of the investigated electrospinning parameters on deposition rate and fiber accumulation. Therefore, thickness was not additionally normalized to deposited mass, as thickness itself was considered a process-dependent response variable. Nevertheless, the potential contribution of mass differences arising from variations in flow rate and needle gauge is acknowledged as a limitation of the study.

In this study, a digital caliper (Mitutoyo micrometer, Kvalifix Kft, Budapest, Hungary) was utilized to measure the thickness of the nanofiber matrices. Thickness measurements were performed in triplicate on single-layer samples on aluminum foil, and the average was calculated. In all cases, the basal aluminum carrier layer was included in the measurement. The procedure followed previously reported methods [20].

#### 2.3.5. Water Uptake Capacity

Swelling measures the amount of water the mat can absorb and how the structure changes when hydrated. Mats with higher swelling capacity can absorb exudates and maintain a moist healing microenvironment.

Using slightly modified established protocols, the water absorption capacity of electrospun nanofiber matrices was determined [20]. 10 × 10 mm rectangular samples were cut and weighed to determine their initial dry mass (W_0_) using an analytical balance (Ohaus, Pioneer, Merck, Budapest, Hungary). Each sample was immersed in 2 mL of MilliQ water and incubated at room temperature for 8 h. After 8 h, the samples were removed, gently blotted to eliminate superficial water, and weighed to obtain the swollen mass (Wt_n_). Water uptake was calculated using the equation below:

$$Water\;uptake\;(\%)\;=\;[({\mathrm{Wt}}_{n}/W_{0})-1]\times 100$$

All the experiments were conducted in triplicates with results exhibited as mean ± standard deviation (SD).

To reduce the influence of differences in deposited material quantity, swelling experiments were performed on specimens of identical dimensions (10 × 10 mm), and water uptake was expressed relative to the initial dry mass of each sample according to the equation. This mass-based normalization enables direct comparison of hydration behavior among nanofiber mats with different thicknesses and deposited masses.

#### 2.3.6. Thermogravimetric (TGA) Analysis

The thermal stability of the obtained compounds was investigated using a TG 209 F1 Libra thermogravimetric analyzer (NETZSCH, Selb, Germany). The measurements were carried out over a temperature range from 25 to 1000 °C under a controlled atmosphere, using nitrogen (10 mL/min) and air (25 mL/min) as purge gases. Samples with a mass of approximately 5–10 mg were placed in alumina (Al_2_O_3_) crucibles. The change in sample mass was continuously recorded as a function of temperature to evaluate decomposition behavior and thermal resistance.

#### 2.3.7. Differential Scanning Calorimetry (DSC) Analysis

The thermal behavior of the samples was analyzed using a differential scanning calorimeter under a nitrogen atmosphere. Approximately 10 mg of each sample was sealed in aluminum crucibles prior to measurement. The analysis was performed using a heating—cooling—heating cycle: samples were initially heated from −50 to 200 °C, subsequently cooled to −50 °C, and then reheated to 200 °C. All heating and cooling steps were carried out at a rate of 10 °C/min.

The optimization based on simultaneous consideration of (1) minimum fiber diameter, (2) low diameter variability, (3) high porosity, (4) stable Taylor cone formation and (5) acceptable swelling behavior. This multi-response optimization strategy can be evaluated quantitatively.

### 2.4. Statistical Analysis

Statistical analysis was performed using ‘R’ software (version 4.5.2 https://www.R-project.org/) [21]. Before conducting the analyzes, the normality of the data was assessed using the Shapiro–Wilk test, which indicated that not all data categories were normally distributed (*p* < 0.05). Therefore, non-parametric statistical methods were employed for further analysis. Spearman’s rank correlation analysis was used to assess relationships among measured nanofiber properties. Kruskal–Wallis tests were applied to evaluate the effects of input parameters (three levels were applied for each parameter) on the measured properties of the produced nanofibers (*p* < 0.05). These tests were followed by Mann–Whitney U post hoc tests. Multiple linear regression models were constructed to analyze the effects of all input parameters together on the properties of the nanofibers. Temperature and humidity values measured during the fabrication of the nanofibers were incorporated into the model to assess their potential effects as well.

## 3. Results

### 3.1. SEM Image Analysis

**Figure 2:**
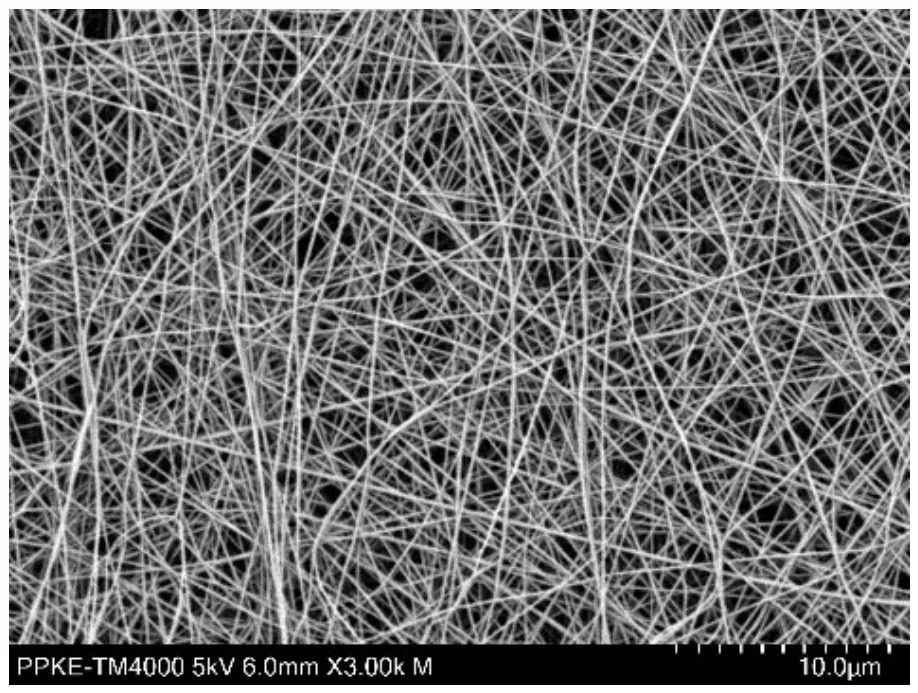
Representative SEM micrograph of a gold-coated electrospun PVA mat, illustrating low variations in fiber diameter, uniformity, and no defect formation (SEM parameters: 5 kV, 6.00 mm, ×3.00 k Magnification, 10 mm scale). The complete collection of SEM images is presented in Figures S1 and S2 in the Supplementary Materials.

Regarding morphological consistency and fiber quality in BBD I, SEM micrographs in Supplementary Figure S1 reveal that most nanofibers fabricated under BBD I conditions exhibit uniform, bead-free morphology, indicating that the selected parameter ranges were appropriate for stable spinning. Some illustrative SEM images are shown in Figure 2. The overall fiber diameters fall within ~198–218 nm (Table 3), confirming that 8% PVA enables the formation of nanoscale fibers at relatively low voltages and flow rates.

Subtle morphological variations are visible across runs. (1) The higher flow rate (Q = 1) in combination with higher voltage (V = 1), e.g., Run 4, produces slightly thinner and more uniform fibers (198.9 ± 3.6 nm), likely because the stronger electric field offsets increased mass flow. (2) Furthermore, the low voltage–low flow combinations (Runs 1–2) yield somewhat larger fibers (~207–211 nm), linking reduced stretching forces to increased diameter.

The effect of needle gauge on fiber morphology (BBD II) can be seen in Supplementary Figure S2. SEM images highlight noticeable differences when needle gauge is included as a factor: (1) Larger needle diameter (16G) tends to increase fiber diameter (e.g., Runs 8, 12, 15), likely due to an increased initial jet volume. (2) Finer needles (20G) generally produce thinner fibers and smoother morphology (Runs 1, 3), consistent with lower initial jet cross section.

This confirms that needle geometry plays a non-negligible role in both jet stability and fiber formation, a trend supported by quantitative results in Table 4.

**Figure 3:**
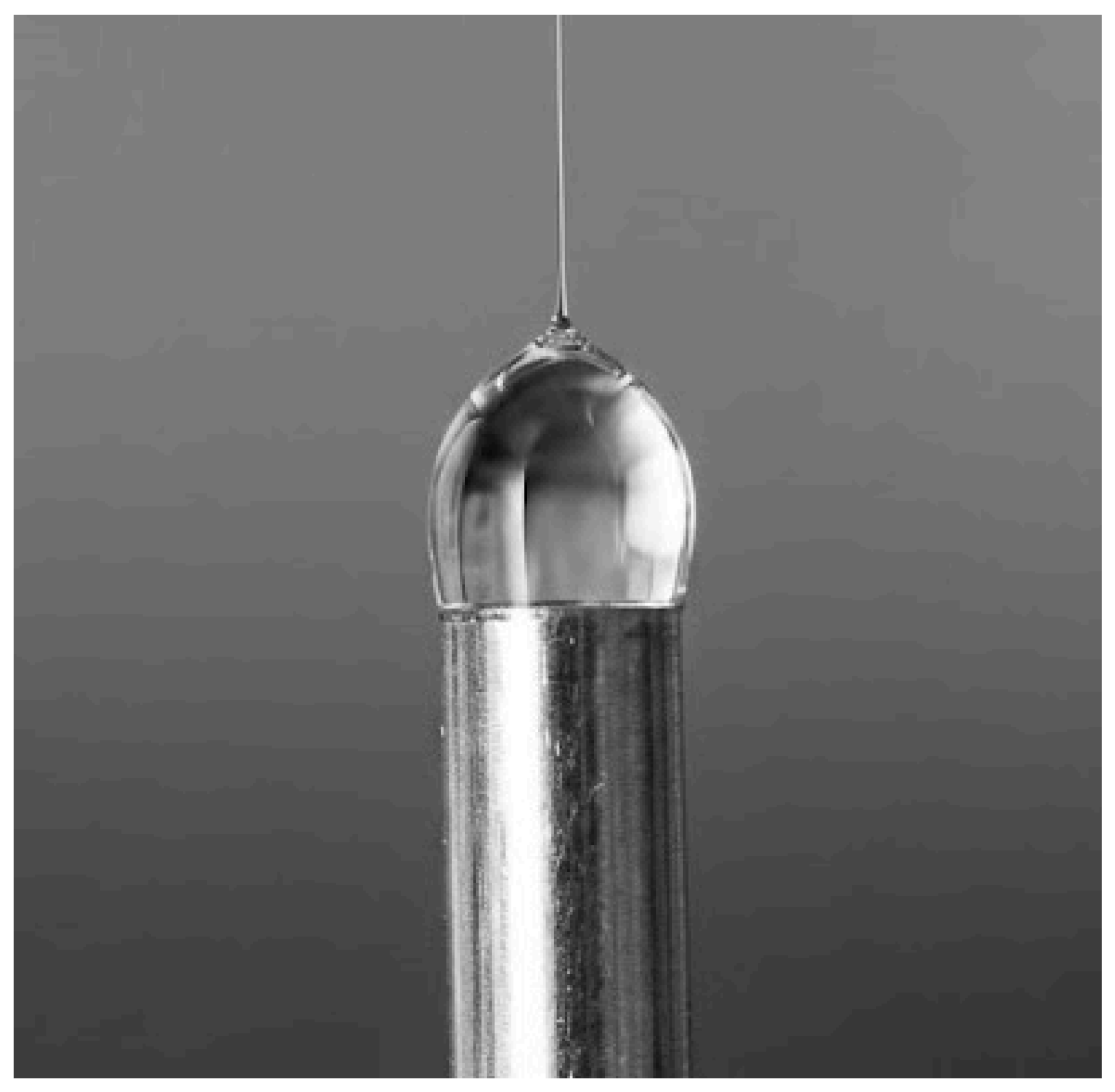
Representative Taylor cone images (rate 4) reflecting qualitative stability of the process and the end product. The complete collection of Taylor cone images is presented in Figures S3 and S4 in the Supplementary Materials.

The Taylor cone images in Supplementary Figures S3 and S4 show a strong alignment between cone stability and successful fiber formation. Some representative high power pictures on Taylor cone formation are presented in Figure 3. Conditions producing Rating 4 (very stable) cones correspond to the most uniform fiber morphologies in SEM. While the lower ratings (1–2) often align with runs that produced minor defects (e.g., slight diameter variability or localized fusing), suggesting insufficient or unstable electrostatic stretching.

The Taylor cone rating therefore serves as a reliable qualitative indicator for identifying optimal electrospinning parameter zones and process robustness.

### 3.2. Mean Fiber Diameter, Uniformity, and Porosity Analysis

The limited overall variation shown in Table 3 demonstrates that the selected parameter window was appropriate. Across all BBD I runs (Table 3), mean diameters remain within a narrow window (~199–218 nm), demonstrating that the selected voltage (15–20 kV), flow rate (0.2–0.5 μL/h), and distance (8–16 cm) ranges keep the spinning process stable and the Box–Behnken approach effectively limited experimental variation and avoided extreme, defect prone regimes.

Supplementary Figure S5 and Table 3 demonstrates, that higher voltage (V) tends to slightly reduce diameter at low flow rates but increases it at high flow rates, indicating an interaction between V and Q. The higher flow rate (Q) generally increases fiber diameter unless compensated by high voltage, and the longer distance (D) produces marginally more variable fibers (e.g., Runs 7, 9), likely due to increased jet path instability.

Overall variation is limited, but these small shifts are consistent with classical electrospinning theory.

Based on the results in Table 4 and Supplementary Figure S6, needle gauge significantly influences fiber diameter (BBD II): 16G needles consistently yield the thickest fibers (e.g., Runs 8, 12, 15, all >220 nm), 20G needles give the smallest diameters (~203–206 nm) and 18G needles (already assessed in BBD I) sit between these extremes.

This confirms that the mechanical factor of the needle orifice significantly modifies the initial jet profile and thus final fiber geometry.

**Figure 4:**
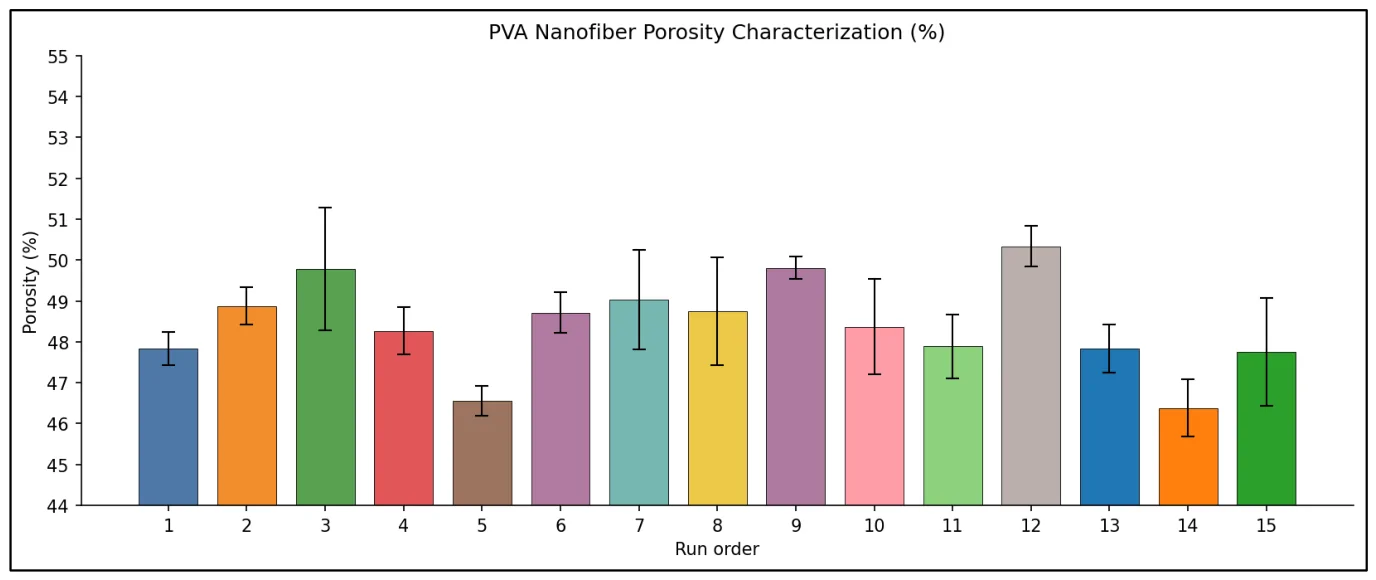
Porosity of electrospun PVA nanofiber mats fabricated under BBD-I conditions, calculated from SEM image analysis (mean ± SD, n = 3).

**Figure 5:**
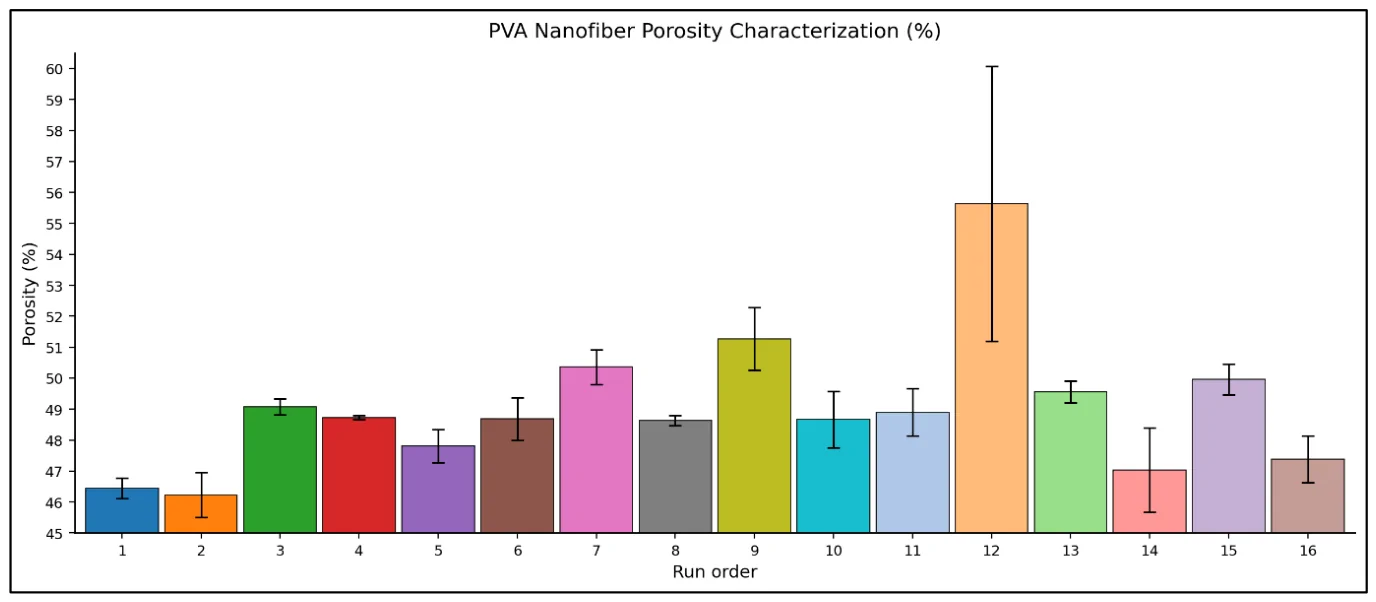
Porosity of electrospun PVA nanofiber mats fabricated under BBD-II conditions, calculated from SEM image analysis (mean ± SD, n = 3).

Porosity values in Figure 4 and Figure 5 reflect that the higher porosity for finer fibers, is consistent with increased surface area and reduced packing density. Runs characterized by larger fiber diameters exhibited reduced porosity (e.g., high flow, large needle) show reduced porosity, aligning with denser fiber packing and greater average fiber thickness.

Thus, porosity and fiber diameter exhibit an inverse relationship, reinforcing their coupled nature. In summary, porosity trends mirror fiber diameter behavior.

### 3.3. Layer Thickness and Swelling Evaluation

**Figure 6:**
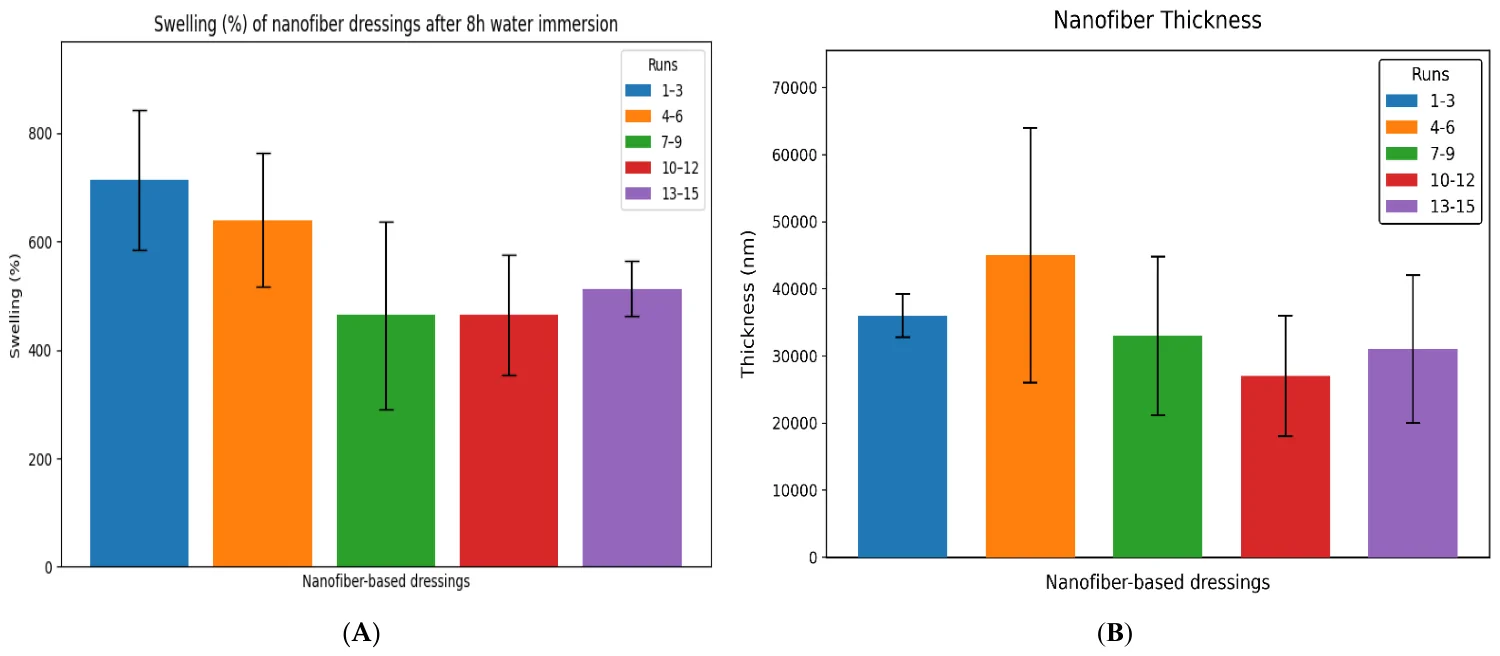
(**A**) Swelling capacity of **BBD-I** PVA nanofiber mats after 8 h water immersion (mean ± SD, n = 3). (**B**) Thickness of electrospun PVA nanofiber mats fabricated under BBD-I conditions (mean ± SD, n = 3).

**Figure 7:**
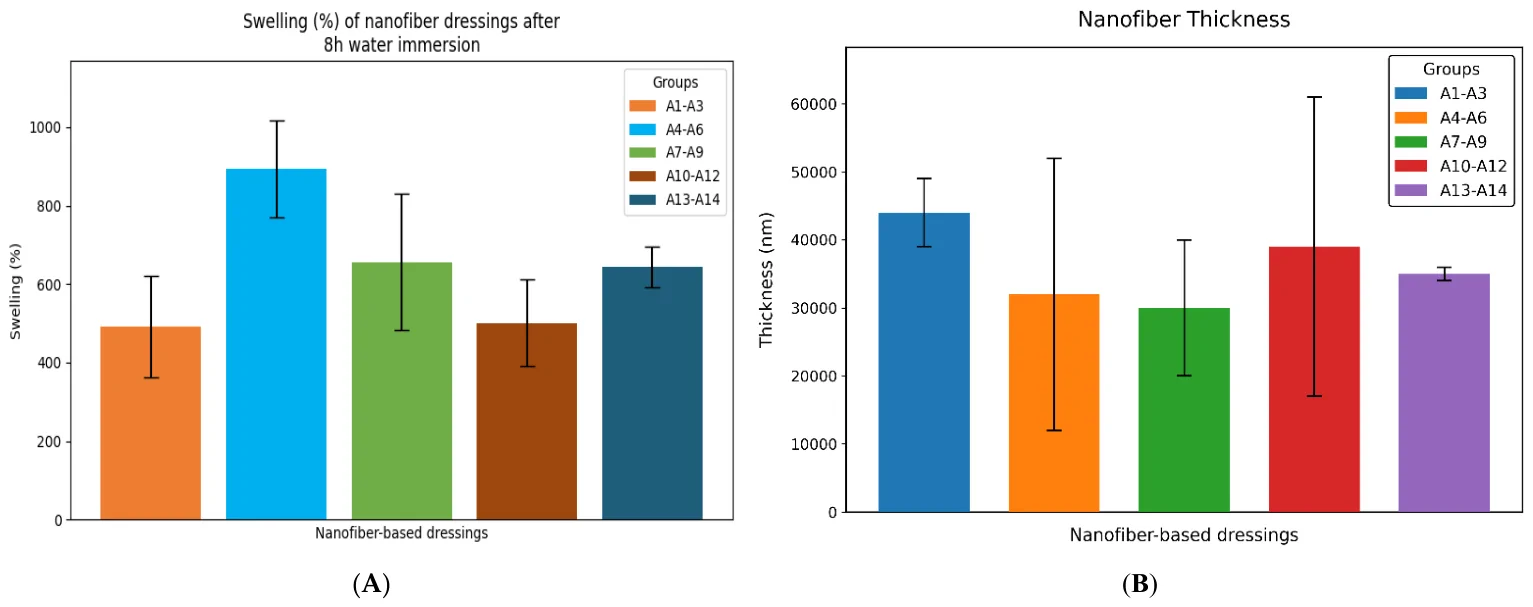
(**A**) Swelling capacity of **BBD-II** PVA nanofiber mats after 8 h water immersion (mean ± SD) n = 3. (**B**) Thickness of electrospun PVA nanofiber mats fabricated under BBD-II conditions (mean ± SD), n = 3.

The layer thickness reflects deposition behavior and the influence of parameters. In Figure 6B and Figure 7B, mat thickness trends correspond to higher flow rate (Q = 1) → thicker mats due to increased polymer delivery, larger needle gauge (16G) → thicker layers in BBD II, consistent with higher mass throughput per unit time and shorter spinning distance (D = −1) → occasionally thicker, less spread-out deposition areas.

The strong agreement between deposition parameters and mat thickness confirms predictable control over macroscopic membrane structure.

Based on the results in Figure 6A and Figure 7A, swelling capacity depends on fiber architecture and mat thickness. Higher swelling can be seen in thinner, more porous mats, where water penetrates more readily. On the other hand, lower swelling is observed in thicker mats, where compact packing and lower porosity hinder diffusion. In BBD II, the needle-size effect is also evident. In 20G needle we can see higher swelling, due to thinner fibers and increased void fraction, while in 16G needle lower swelling, aligning with denser, thicker mats was detected.

These results align with known relationships between PVA nanofiber mat microstructure and hydration behavior.

The statistical evaluation of the relationships between the manufacturing parameters and the properties of the end product is presented in Figure 8, Figure 9, Figure 10, Figure 11, Figure 12, Figure 13, Figure 14 and Figure 15.

**Figure 10:**
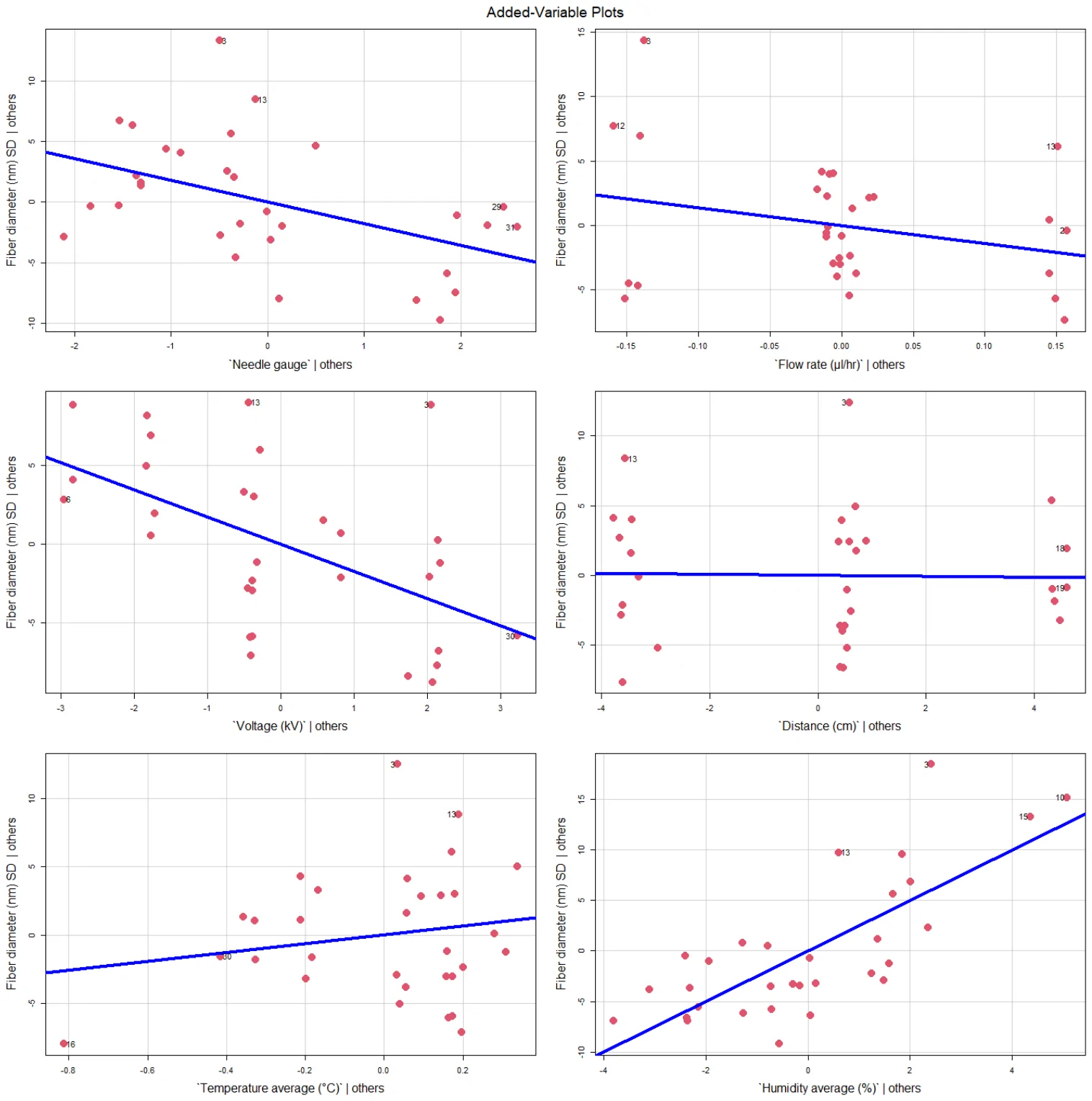
Added variable (partial regression) plots from the multiple linear regression model (n = 31) of fiber uniformity (diameter variability (SD)) using needle gauge, flow rate, voltage, distance input parameters and temperature and humidity as measured additional environmental parameters. Blue lines indicate the fitted trend lines of the parameter of interest after controlling for all other parameters. Red dots: individual data.

**Figure 11:**
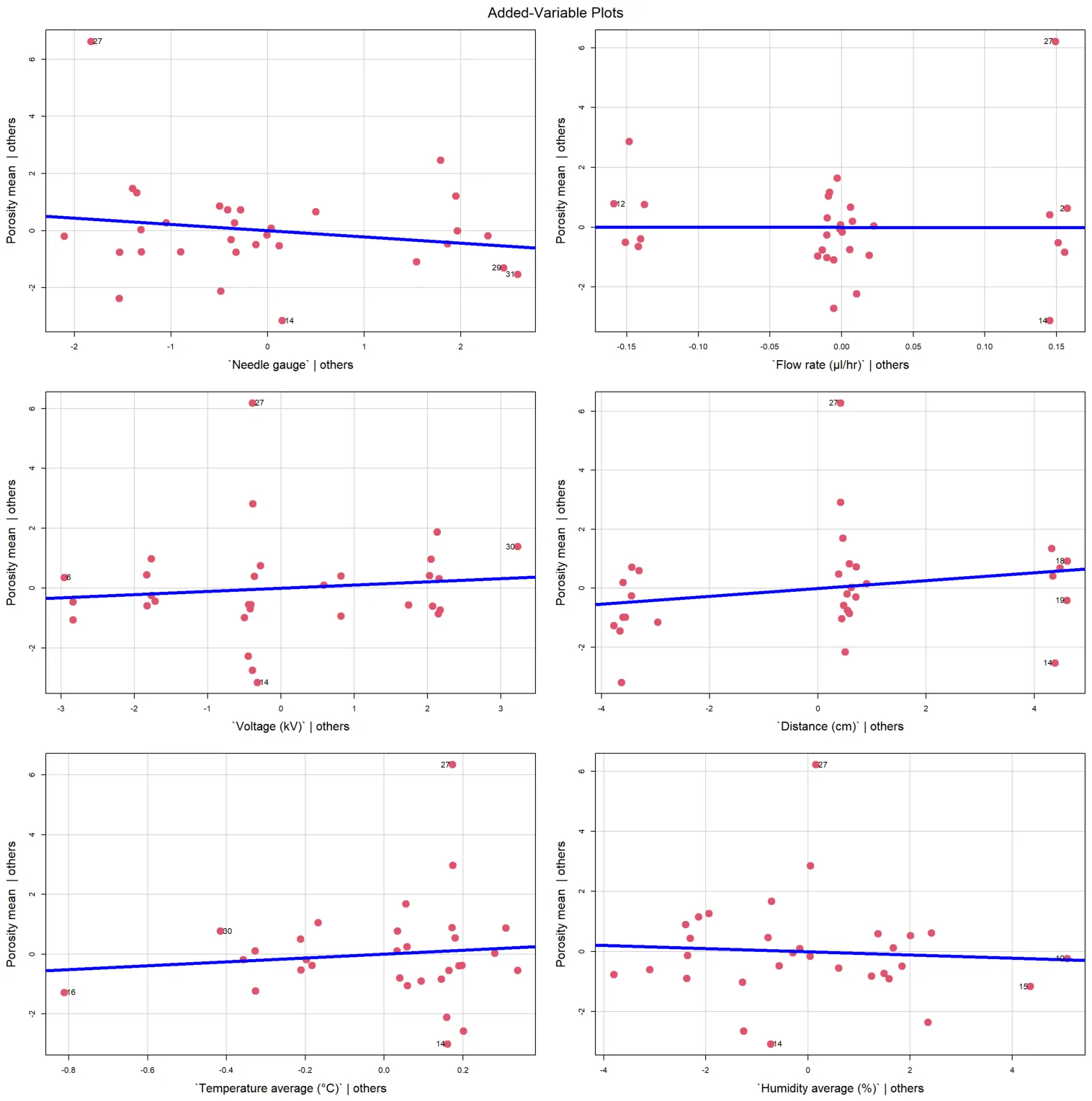
Added variable (partial regression) plots from the multiple linear regression model (n = 31) of mean porosity using needle gauge, flow rate, voltage, distance input parameters and temperature and humidity as measured additional environmental parameters. Blue lines indicate the fitted trend lines of the parameter of interest after controlling for all other parameters. Red dots: individual data.

**Figure 12:**
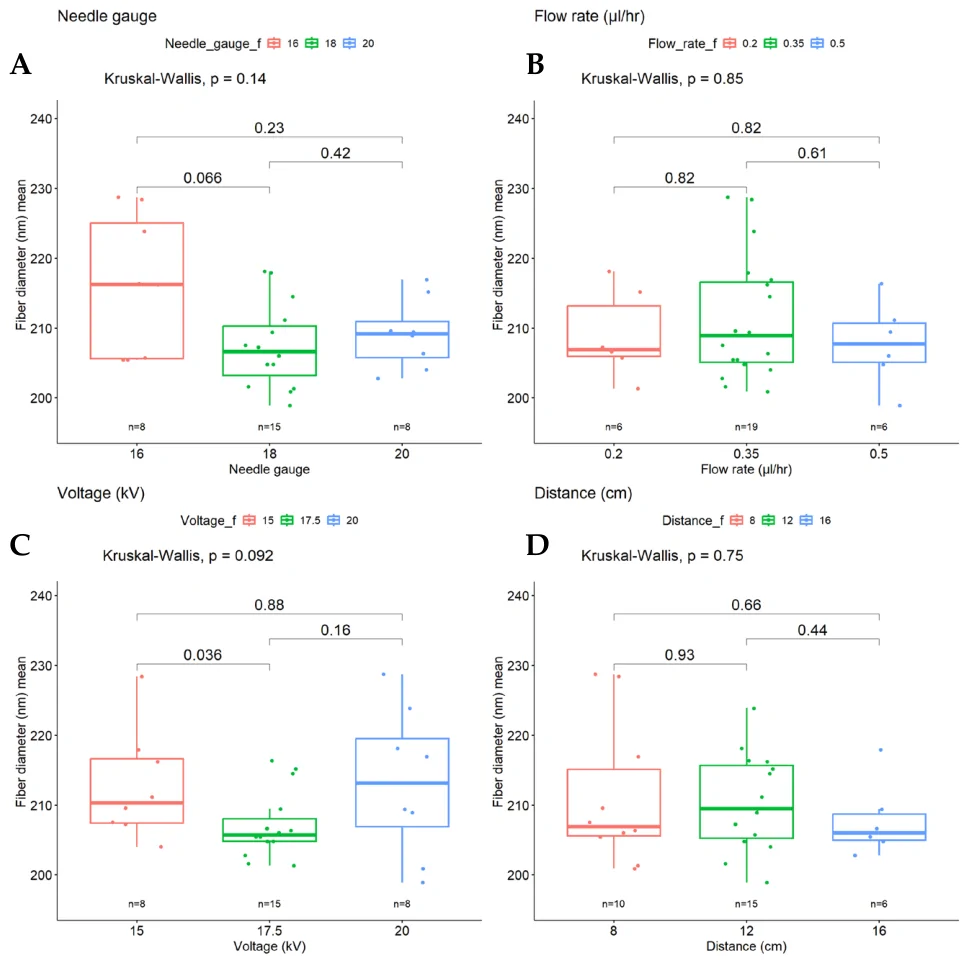
Comparison of mean fiber diameters for different levels of input parameters ((**A**): needle gauge, (**B**): flow rate, (**C**): voltage, (**D**): distance) using Kruskal–Wallis test and Mann–Whitney U post hoc tests. *p*-values are noted above, and group sizes are noted below the boxplots.

**Figure 13:**
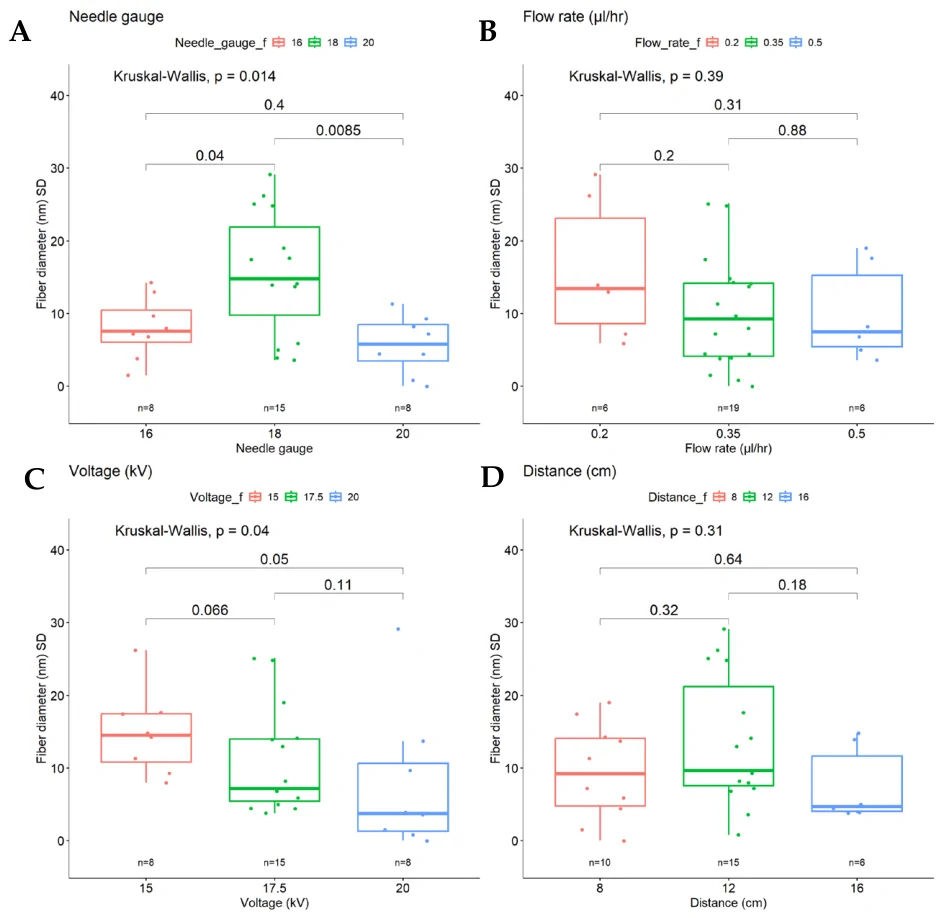
Comparison of fiber uniformity (diameter variabilities (SD)) for different levels of input parameters ((**A**): needle gauge, (**B**): flow rate, (**C**): voltage, (**D**): distance) using Kruskal–Wallis test and Man-Whitney U post hoc tests. *p*-values are noted above, and group sizes are noted below the boxplots.

**Figure 14:**
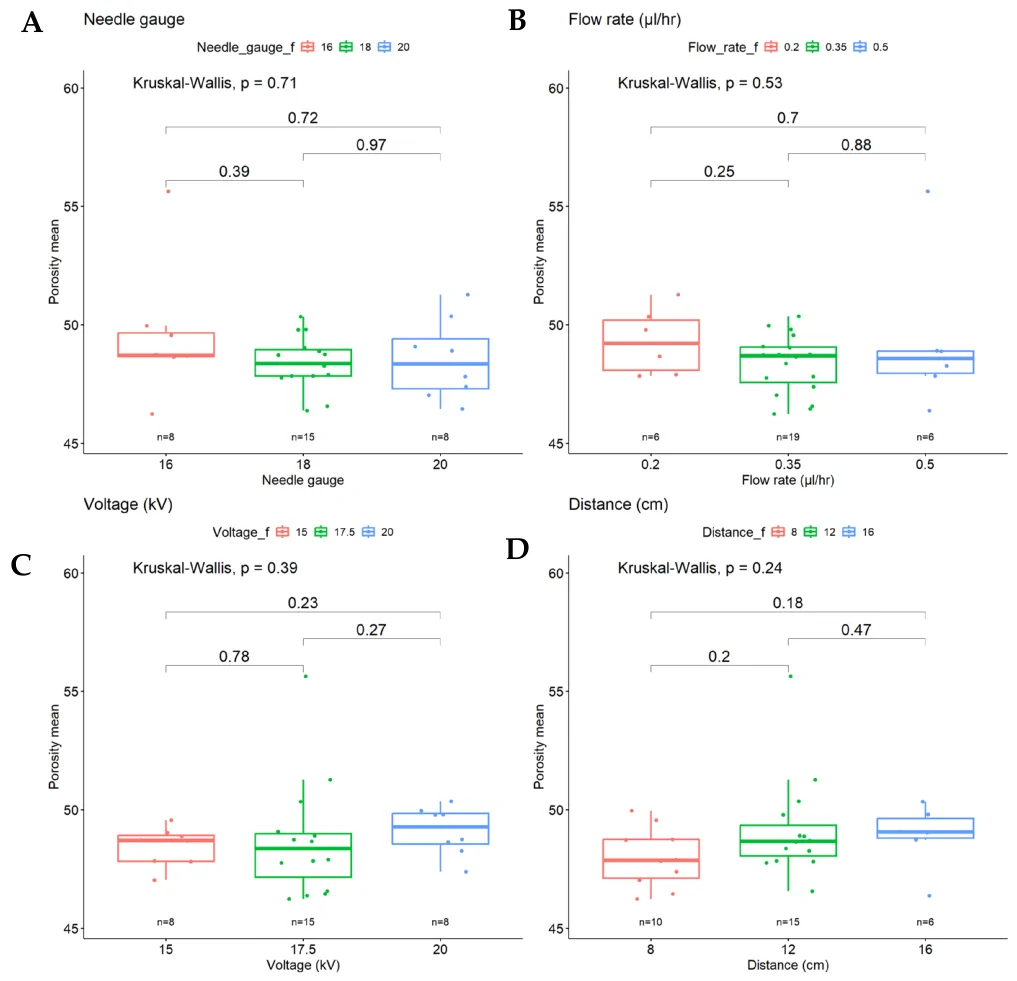
Comparison of mean porosity for different levels of input parameters ((**A**): needle gauge, (**B**): flow rate, (**C**): voltage, (**D**): distance) using Kruskal–Wallis test and Mann–Whitney U post hoc tests. *p*-values are noted above, and group sizes are noted below the boxplots.

The data collectively demonstrated (Figure 10, Figure 11, Figure 12, Figure 13 and Figure 14) the following: 1. Electrospinning was stable across the chosen range, evidenced by consistent Taylor cone ratings, predominantly uniform SEM morphologies, and narrow diameter distributions. 2. Fiber diameter is influenced most strongly by flow rate and needle gauge, with voltage and distance modulating their effects rather than acting independently. 3. Porosity, thickness, and swelling are directly linked to fiber diameter and jet stability, confirming that microstructural tuning enables predictable control of bulk mat performance. 4. The addition of needle gauge as a factor in BBD II substantially improved parameter resolution, revealing effects masked in BBD I.

These findings demonstrate that systematic multiparametric optimization can reliably tailor PVA nanofiber characteristics for wound dressing functionality [22].

**Figure 15:**
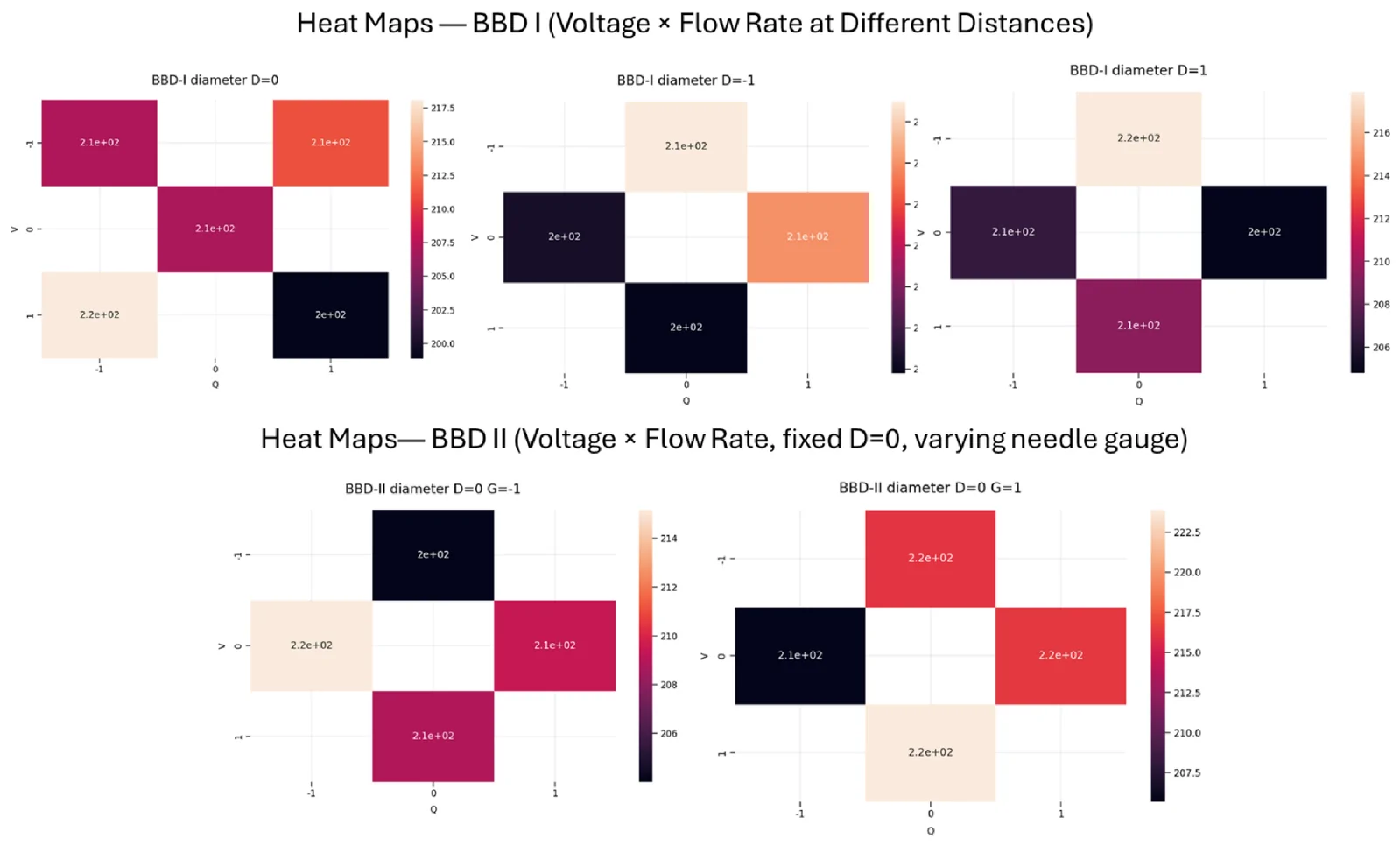
Heat maps of BBD I (connections between voltage–flow rate and spinning distances) and BBD II (connections between voltage–flow rate and needle gauge at fixed spinning distance D = 0) manufacturing designs. Dark color reflects low values; light color reflects high values.

As it is demonstrated in Figure 15. the needle gauge = –1 (20G), corresponds to the thinnest needle generally leads to thinner fibers. Needle gauge = +1 (16G), shows that large values generally result in thicker fibers.

The heat maps (Figure 15) provide a visual representation of the interactions among electrospinning parameters; however, interpretation was supported by the multiple linear regression analysis. The regression models demonstrated that needle gauge and flow rate exerted the strongest effects on fiber diameter, whereas voltage and spinning distance mainly modified the effects of the primary factors. The largest interaction was observed between flow rate and voltage, consistent with the heat-map gradients, indicating that increasing voltage partially compensated for the diameter-increasing effect of higher flow rates. In contrast, spinning distance exhibited comparatively weaker interaction effects. The heat maps therefore complement the regression analysis by revealing parameter combinations associated with similar responses and illustrating interaction patterns that are less obvious from individual regression coefficients alone.

### 3.4. Thermogravimetric (TGA) Analysis Results

Thermogravimetric analysis was performed to evaluate the thermal stability of electrospun PVA nanofiber mats prepared under BBD-I and BBD-II conditions. The results are presented in Table 5, Table 6 and Table 7 as mean ± SD obtained from two or three independent measurements. Isolated outliers were excluded only when one replicate clearly deviated from two closely matching measurements and was accompanied by inconsistent peak assignment, indicating experimental error rather than genuine thermal behavior. Exclusion of these isolated values did not influence the overall interpretation of the degradation profiles.

All tested PVA nanofiber samples exhibited a multistep degradation profile. The first characteristic temperature (T_onset_) reflects the onset of thermal decomposition, whereas T_max1_ is generally attributed to the main degradation stage of PVA involving dehydration and chain scission reactions. T_max2_ is associated with the decomposition of the remaining carbonaceous structures.

For BBD-I samples, T_onset_ values ranged from 229.3 ± 7.2 °C to 280.0 ± 4.5 °C, indicating that the onset of thermal degradation was generally above 220 °C. Although moderate differences in T_onset_ were observed among individual samples, no systematic dependence on any single electrospinning parameter could be identified. In contrast, T_max1_ remained comparatively stable across the design, with most samples decomposing within a narrow temperature interval close to 270–275 °C. This low variability indicates that the main PVA degradation mechanism was not substantially altered within the investigated ranges of voltage, flow rate, and spinning distance. Consequently, optimization of the electrospinning process can be achieved without compromising the inherent thermal stability of the polymer.

The second high-temperature degradation event (T_max2_) occurred mostly between approximately 440 and 467 °C. The variability observed for T_max2_ most likely reflects differences in the morphology and packing of the electrospun mats rather than changes in the chemical composition of PVA. Overall, BBD-I samples displayed a mean T_onset_ of 247.0 ± 11.7, T_max1_ of 271.6 ± 6.4, and T_max2_ of 457.5 ± 10.7. These findings indicate that variations in voltage, flow rate, and spinning distance primarily influenced the morphology of the electrospun mats rather than the intrinsic thermal stability of PVA.

The BBD-II set, which additionally included needle gauge as a process variable, showed slightly lower and more dispersed T_onset_ values than BBD-I. The onset temperatures ranged from 225.3 ± 5.1 °C to 248.7 ± 3.3 °C, with an overall mean of 233.9 ± 9.1. The BBD-II design exhibited slightly greater variability in T_onset_ values than BBD-I, which may be associated with the additional influence of needle geometry on fiber architecture and mat organization, together with experimental variability inherent to electrospinning processes.

Despite this increased variability, the main degradation temperature remained centered around the same region as in BBD-I. The overall T_max1_ value for BBD-II was 269.2 ± 10.5, confirming that the fundamental degradation pathway of PVA was retained. T_max2_ values showed greater dispersion, with an overall value of 452.4 ± 12.8, likely reflecting differences in the morphology and organization of the carbonaceous residue formed during degradation. Samples 10–12 and 15 exhibited lower T_max2_ values or higher SD, suggesting less homogeneous thermal behavior in selected mats. Although individual samples exhibited moderate differences in T_onset_, no systematic dependence on any single electrospinning parameter was observed. This suggests that the influence of electrospinning parameters on thermal behavior is secondary to their effects on fiber morphology and mat architecture.

The multistep degradation profile observed in this study agrees well with previous reports for electrospun PVA, where the initial degradation stage is attributed to dehydration and elimination reactions, followed by decomposition of the polymer backbone and carbonaceous residues [11,23,24].

Overall, the relatively small differences observed in T_onset_ and T_max1_ indicate that the investigated electrospinning parameters had only a limited influence on the intrinsic thermal stability of PVA. Instead, these parameters primarily affected the physical organization of the electrospun mats, as reflected by the greater variability observed for T_max2_. These observations are consistent with the SEM and DSC analyses, which likewise demonstrated that electrospinning conditions predominantly modified fiber morphology and structural organization without altering the fundamental thermal degradation pathway of the polymer.

### 3.5. Differential Scanning Calorimetry (DSC)

The thermal transitions of electrospun PVA nanofiber mats prepared according to the BBD-I experimental design were evaluated using differential scanning calorimetry. The resulting parameters are summarized in Table 8 and Table 9. The interpretation focused primarily on reproducible transitions observed across replicate measurements, whereas thermal events detected in only one replicate were treated as descriptive observations and were not used to establish general trends.

All BBD-I samples exhibited a reproducible glass transition temperature (Tg) within a narrow range of 68.9–70.2 °C. The relatively small differences among the samples indicate that variations in applied voltage, flow rate, and spinning distance had only a limited effect on the segmental mobility of the amorphous PVA phase. However, because the Tg region may be influenced by residual moisture and partially overlap with broad low-temperature endothermic effects, small differences between individual samples should not be overinterpreted.

A broad low-temperature endothermic event, designated Tp1, was detected between approximately 52 and 60 °C. This effect may be associated with the release of residual moisture and relaxation of the hydrophilic PVA matrix. Its position partially overlapped with the glass-transition region, and the corresponding ΔH1 values showed considerable variability. These results suggest differences in sample hydration and local polymer-chain organization; nevertheless, the variability of this event prevents a direct attribution to individual electrospinning parameters.

Several samples showed an additional event, designated Tp2, between approximately 74 and 112 °C. In most cases, this transition was detected in only one replicate and was therefore not considered a reproducible characteristic of the corresponding nanofiber mat. It may reflect heterogeneous moisture distribution, localized molecular rearrangements, or minor baseline-related effects. Accordingly, Tp2 and ΔH2 were interpreted only descriptively and were not used for quantitative comparison among the experimental conditions.

The most reproducible high-temperature endothermic event, Tp3, was observed at approximately 191–193 °C in nearly all BBD-I samples (Table 8). This transition is consistent with melting of crystalline PVA domains and agrees with previously reported melting temperatures for partially hydrolyzed PVA. Its narrow temperature range indicates that the investigated electrospinning parameters did not substantially change the thermal conditions required for melting of the ordered polymer regions.

The measured ΔH3 values were relatively low and variable. Although this may suggest a limited contribution of ordered domains in the electrospun mats, the degree of crystallinity cannot be determined reliably from these values alone without normalization to dry polymer mass and an appropriate reference enthalpy for fully crystalline PVA. The results should therefore be interpreted as evidence of similar melting behavior rather than as a quantitative measure of crystallinity.

Overall, the reproducibility of Tg and Tp3 indicates that the BBD-I process variables predominantly influenced fiber morphology and mat architecture rather than the fundamental thermal transitions of PVA. This conclusion is consistent with the TGA and SEM results, which similarly indicate preservation of the principal polymer characteristics across the investigated processing window.

The thermal transitions of the PVA nanofiber mats prepared according to the BBD-II experimental design are summarized in Table 9. As in the BBD-I analysis, emphasis was placed on reproducible thermal events, whereas transitions detected in only one measurement were considered descriptive.

The glass-transition temperatures determined during the first and second heating cycles were observed at approximately 60–65 °C and 68–71 °C, respectively. The shift toward higher Tg values during the second heating cycle may result from removal of residual moisture and elimination of the previous thermal history during the first heating step. Because water acts as a plasticizer for PVA, its partial removal decreases polymer-chain mobility and may increase the glass-transition temperature observed during reheating. The narrow Tg range within each heating cycle indicates that the investigated electrospinning parameters, including needle gauge, had only a limited influence on the amorphous-phase mobility of PVA.

A broad low-temperature endothermic event, Tp1, was observed between approximately 49 and 61 °C. This event may be related primarily to residual moisture release and relaxation of the hydrophilic polymer matrix. The considerable variability in ΔH1 indicates differences in sample hydration or local mat organization. However, no systematic relationship between ΔH1 and an individual BBD-II process variable could be established.

An additional Tp2 event was observed in selected samples between approximately 72 and 88 °C. Because this event was absent from several samples and was frequently detected in only one replicate, it cannot be regarded as a characteristic transition of the BBD-II nanofiber mats. It may arise from heterogeneous water binding, localized polymer rearrangements, or minor differences in thermal history. Consequently, neither the occurrence nor the enthalpy of Tp2 was used to rank the samples or assess the effects of individual electrospinning parameters.

The high-temperature endothermic peak Tp3 was consistently observed between approximately 190 and 193 °C. This transition is consistent with melting of ordered PVA domains. Its narrow temperature range indicates that the inclusion of needle gauge as an experimental factor in BBD-II provided additional insight into the effects of spinneret geometry. Needle gauge did not substantially alter the melting temperature of the polymer. Nevertheless, the similarity of Tp3 values alone does not demonstrate an identical degree of crystallinity, which would require quantitative analysis of normalized melting enthalpies and complementary structural characterization, such as XRD or WAXS.

Overall, the BBD-II samples showed comparable principal thermal transitions. The process variables, including needle gauge, appear to have affected hydration, local polymer-chain organization, and nanofiber-mat architecture more strongly than the fundamental melting behavior of PVA. These findings are consistent with the TGA results and support the conclusion that the investigated electrospinning conditions preserved the principal thermal characteristics of the polymer.

The observed glass-transition, moisture-related, and melting events are generally consistent with previous DSC studies of PVA reported by Holland and Hay, Mansur et al., and Gaaz et al. [11,23,24]. However, assignments of weak or non-reproducible secondary events remain tentative because complementary structural analyses were not performed.

## 4. Discussion

The present study investigated the influence of applied voltage, flow rate, spinning distance, and needle gauge on the fabrication of electrospun PVA nanofibers intended for wound-dressing applications. By combining two Box–Behnken experimental designs with morphological, physicochemical, and thermal characterization, the work provides insights into the electrohydrodynamic mechanisms governing fiber formation and their impact on the functional properties of the resulting nanofiber mats.

Electrospinning is governed by a complex balance between electrostatic forces, surface tension, viscoelastic resistance, and solvent evaporation dynamics. When a sufficiently high electric field is applied to a polymer solution droplet, electrostatic forces overcome surface tension, resulting in the formation of a Taylor cone and the ejection of a charged polymer jet [7,14]. As the jet accelerates toward the collector, electrostatic repulsion among surface charges induces extensive stretching and thinning, ultimately producing nanometer-scale fibers [5,8].

The relatively narrow fiber diameter range obtained in the present study (198–229 nm) indicates that the selected process window maintained a favorable balance between electric-field-driven stretching and the viscoelastic resistance of the PVA solution. PVA is known to possess sufficient molecular-chain entanglement to stabilize the electrospinning jet and suppress bead formation at appropriate concentrations [11,25]. The bead-free morphologies observed across most experimental conditions therefore suggest that the chosen 8% PVA formulation provided adequate viscoelasticity for continuous fiber formation. Similar diameter ranges have recently been reported for optimized electrospun PVA systems intended for biomedical applications, where fiber diameters between 180 and 350 nm were considered advantageous for mimicking extracellular matrix architecture [2,13]. The interaction between applied voltage and flow rate is one of the most important determinants of electrospinning performance. Increasing voltage increases the electrostatic force acting on the polymer droplet, promoting jet stretching and reducing fiber diameter. However, this effect is highly dependent on the rate at which the solution is supplied to the needle tip [5,14].

In the present study, higher voltages reduced fiber diameter only under low-flow conditions, whereas the diameter-increasing effect of higher flow rates could partially offset voltage-induced thinning. Mechanistically, higher flow rates increase the volume of solution available for jet formation, resulting in larger initial jet diameters and shorter residence times for solvent evaporation. Consequently, the polymer jet experiences less stretching before reaching the collector, producing thicker fibers [7,16].

Recent optimization studies on PVA/PLA and PVA-based nanofibers reported similar behavior, identifying flow rate as one of the strongest predictors of fiber diameter because it directly controls mass throughput and jet volume [7,18]. Our findings are therefore consistent with both theoretical electrohydrodynamic models and contemporary experimental observations.

The inclusion of needle gauge as a factor in BBD-II provided additional insight into spinneret geometry effects. Needle gauge directly influences the size of the polymer droplet at the needle tip and consequently affects the initial geometry of the Taylor cone. Larger-bore needles (16G) produce larger droplets and higher initial jet volumes, whereas smaller needles (20G) generate smaller droplets and finer jets.

This phenomenon was clearly reflected in the observed fiber diameters, where 16G needles consistently produced thicker fibers than 20G needles. Similar trends have recently been described by Fatahian and Erfani (2025) [17] and Mahdian et al. (2026) [18], who demonstrated that spinneret geometry significantly influences fiber diameter even when solution composition remains unchanged. The stronger dependence of fiber diameter on needle gauge observed in the present study further highlights the importance of considering geometric process variables during electrospinning optimization, an aspect that is frequently overlooked in conventional design-of-experiment studies.

Taylor cone stability represents one of the most sensitive indicators of electrospinning process robustness. Stable Taylor cones indicate equilibrium between electrostatic attraction, gravitational forces, surface tension, and viscoelastic resistance. In contrast, unstable or oscillating cones often generate nonuniform jets, bead formation, and fibers with wider diameter distributions [8,14].

The qualitative Taylor cone scoring system used in this study demonstrated strong agreement with SEM-based fiber quality assessment. Experimental runs exhibiting stable Taylor cones (ratings 3–4) consistently yielded smoother and more uniform fibers, whereas lower-rated cones were associated with increased diameter variability. This observation suggests that Taylor cone monitoring may serve as a practical in-process quality-control parameter for future automated electrospinning systems. Recent machine-learning-based electrospinning studies have likewise identified Taylor cone morphology as a promising predictive indicator of fiber quality and manufacturing reproducibility [15,17]. It should be noted that swelling behavior depends not only on average fiber diameter but also on the uniformity of the fibrous network. Under unstable electrospinning conditions, thin fibers may still be produced; however, bead formation, local fiber fusion, and heterogeneous pore architecture can introduce variability in water uptake and dimensional stability. Consequently, stable Taylor cone formation is advantageous not only for controlling fiber diameter but also for achieving reproducible swelling performance.

Solvent evaporation kinetics play a crucial role in determining final fiber morphology and mat architecture. During electrospinning, water evaporation occurs simultaneously with jet stretching and solidification. If solvent removal is insufficient, fibers may reach the collector before complete solidification, resulting in fusion defects or reduced porosity. Conversely, efficient evaporation promotes uniform fiber formation and preservation of interfiber voids [5,25].

The inverse relationship observed between fiber diameter and porosity can be explained by differences in packing density. Thinner fibers generated larger void fractions and more interconnected pore structures, whereas thicker fibers produced denser networks with reduced porosity. Similar relationships have been reported for electrospun PVA, chitosan, and PVA/chitosan composites used in wound care applications [10,26].

In addition, fiber diameter significantly influenced swelling performance. Mats containing thinner fibers exhibited greater water uptake, likely due to their larger specific surface area and more accessible pore networks. Reduced fiber diameter therefore simultaneously increased porosity and fluid absorption capacity. This finding agrees with previous reports demonstrating that electrospun PVA nanofibers with diameters below 300 nm generally exhibit superior hydration behavior compared with thicker microfibrous structures [2,20].

DSC analysis indicated that the investigated electrospinning conditions had only a limited effect on the principal thermal transitions of PVA. The reproducible glass-transition temperatures and melting peaks observed at approximately 190–193 °C suggest broadly comparable amorphous-phase mobility and melting behavior across the investigated processing conditions. However, the similarity of the melting temperatures alone does not demonstrate an identical degree of crystallinity, which would require quantitative analysis of normalized melting enthalpies and complementary structural characterization, such as XRD or WAXS. The weak or non-reproducible secondary thermal events were therefore interpreted only descriptively and were not used to establish process-dependent trends. These observations are generally consistent with previous DSC studies of PVA-based materials [11,23,24].

Thermogravimetric analysis showed degradation onset temperatures above approximately 225 °C for all nanofiber mats, confirming that thermal decomposition occurred far above the temperatures expected during storage, handling, and physiological application. The relatively narrow distribution of the principal degradation temperatures indicates that the process variables investigated had only a limited effect on the intrinsic thermal resistance of PVA. The greater variability observed for selected high-temperature events may reflect differences in fiber packing, sample heterogeneity, and the physical organization of the residue, rather than changes in polymer composition. Overall, the comparable multistep degradation profiles suggest preservation of the principal degradation behavior of PVA across the investigated processing window. Similar thermal behavior has been reported for electrospun PVA and PVA-based wound-dressing materials [2,20].

Electrospun wound dressings should ideally combine high porosity, adequate fluid absorption capacity, gas permeability, structural stability, and biomimetic architecture. The nanofiber mats developed in this work exhibited several physicochemical characteristics relevant to these requirements. Fiber diameters ranging from approximately 200 to 230 nm closely resemble the fibrous dimensions of natural extracellular matrix components, which can promote cellular attachment and tissue integration [1,5]. In addition, the observed combination of high porosity and swelling capacity suggests favorable exudate-management potential, which is a critical requirement for modern wound dressings [3,4].

Compared with recent reports, the optimized fibers produced in this study fall within the lower end of the diameter range typically reported for electrospun PVA wound dressings (200–700 nm), potentially providing increased surface area and improved fluid management characteristics [2,13]. Furthermore, all formulations demonstrated thermal stability substantially exceeding expected storage and application temperatures. Nevertheless, morphology and thermal stability alone are insufficient to confirm clinical suitability. Mechanical testing, water-vapor transmission measurements, cytocompatibility studies, antimicrobial performance, and in vitro or in vivo wound-healing assays are still required before definitive conclusions regarding biomedical application can be drawn. Therefore, the present findings should be interpreted as evidence of promising physicochemical characteristics, rather than direct proof of wound-healing efficacy.

A limitation of the present work is that rheological properties, conductivity, and detailed solution-state characterization were not investigated. Although all experiments were performed using identically prepared PVA solutions to minimize variability, future studies should incorporate rheological and physicochemical analyses to better elucidate the mechanistic relationships between solution properties, Taylor cone stability, and fiber formation.

Overall, this study demonstrates that systematic multiparametric optimization can successfully establish process–structure–property relationships in electrospun PVA systems. The results confirm that flow rate and needle geometry are the dominant variables controlling fiber morphology, while Taylor cone stability provides a valuable indicator of process reproducibility. These findings contribute to the development of reproducible electrospinning strategies for the fabrication of nanofibrous materials with characteristics relevant to advanced wound-care applications.

## 5. Conclusions

This study demonstrated that electrospinning of PVA nanofibers can be effectively optimized using a multiparametric Box–Behnken design. The main novelties, advantages, and limitations of the present study are summarized below:

(1) The main novelty was the integrated evaluation of applied voltage, flow rate, spinning distance, and needle gauge, combined with Taylor cone stability assessment and comprehensive characterization of morphology, porosity, thickness, swelling behavior, and thermal properties.
(2) Advanced statistical analysis established clear process–structure–property relationships, providing a systematic framework for the optimization of electrospun PVA wound-dressing materials.
(3) A limitation of the study is that Box–Behnken models rely on quadratic response surfaces and may not fully capture highly nonlinear electrospinning phenomena or higher-order parameter interactions. Furthermore, the optimization was performed for a single PVA concentration and within a limited environmental range, restricting the applicability of the optimum conditions to the investigated design space.
(4) Future work should incorporate additional formulation and process variables, including polymer concentration, viscosity, elasticity, conductivity, functional additives, and broader environmental conditions.

The resulting larger datasets would support the application of machine-learning approaches, such as artificial neural networks, random forests, Gaussian-process models, and surrogate-based inverse-design methods [16,18]. These approaches are particularly suitable for describing the complex and nonlinear relationships governing electrospinning processes and have shown strong potential for predicting nanofiber morphology and identifying processing conditions required to achieve target material properties [16,18,27].

Although the optimized nanofibers exhibited promising properties for wound-dressing applications [10,28,29,30,31,32], further mechanical, chemical, release/permeability, biological, microbiological, and wound-healing evaluations are necessary to confirm clinical suitability.

## Figures and Tables

**Figure 8:**
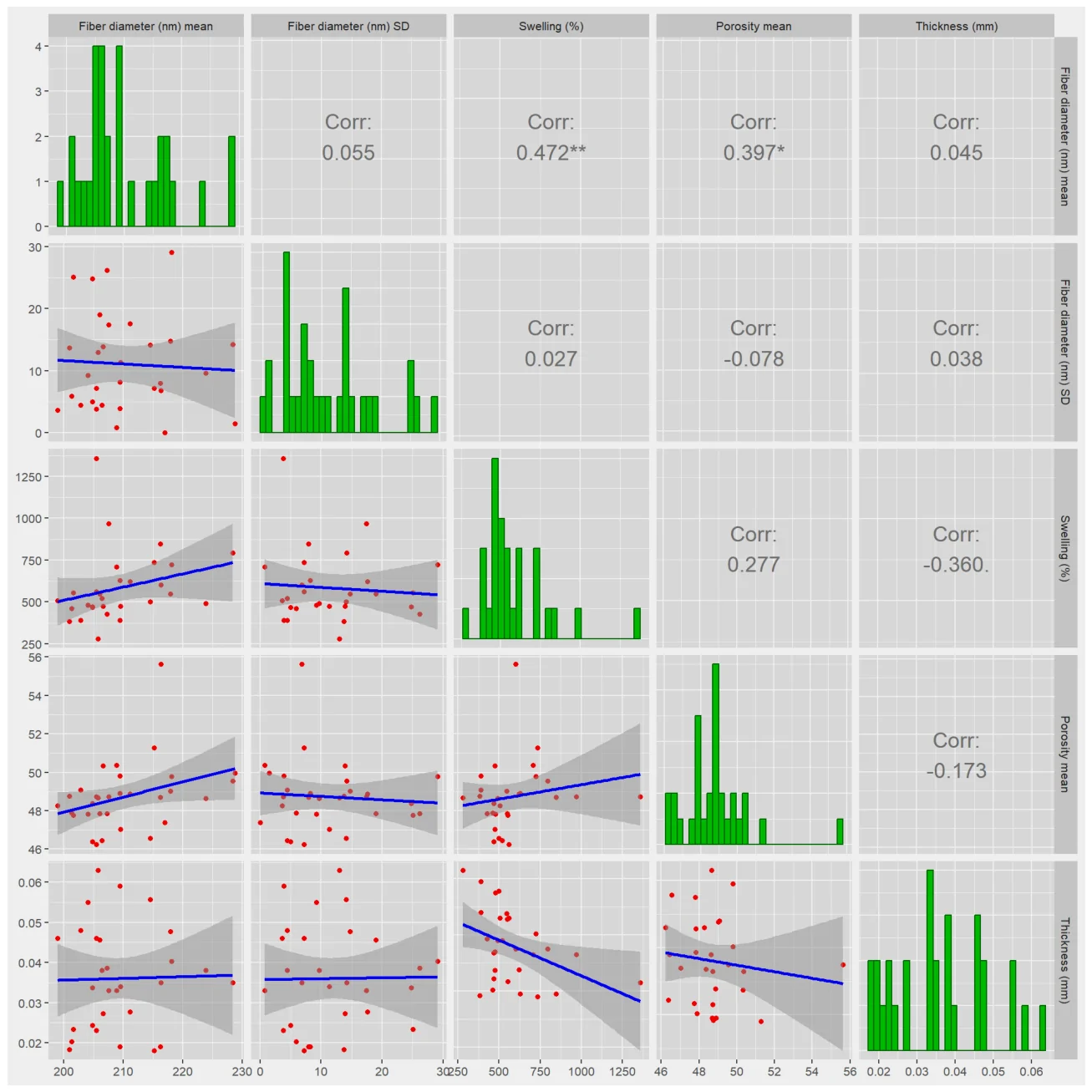
Spearman correlations between five output parameters of the 31 measurements. The plots on the diagonal show the distribution of the parameters; the scatter plots on the lower triangle visualize the relationship between the given parameters with linear trend lines and their confidence intervals; and the upper triangle contains the Spearman correlation coefficients of the given parameters (one asterisk means that the coefficient is significant at the 5% level, two mean that it is significant at the 1% significance level).

**Figure 9:**
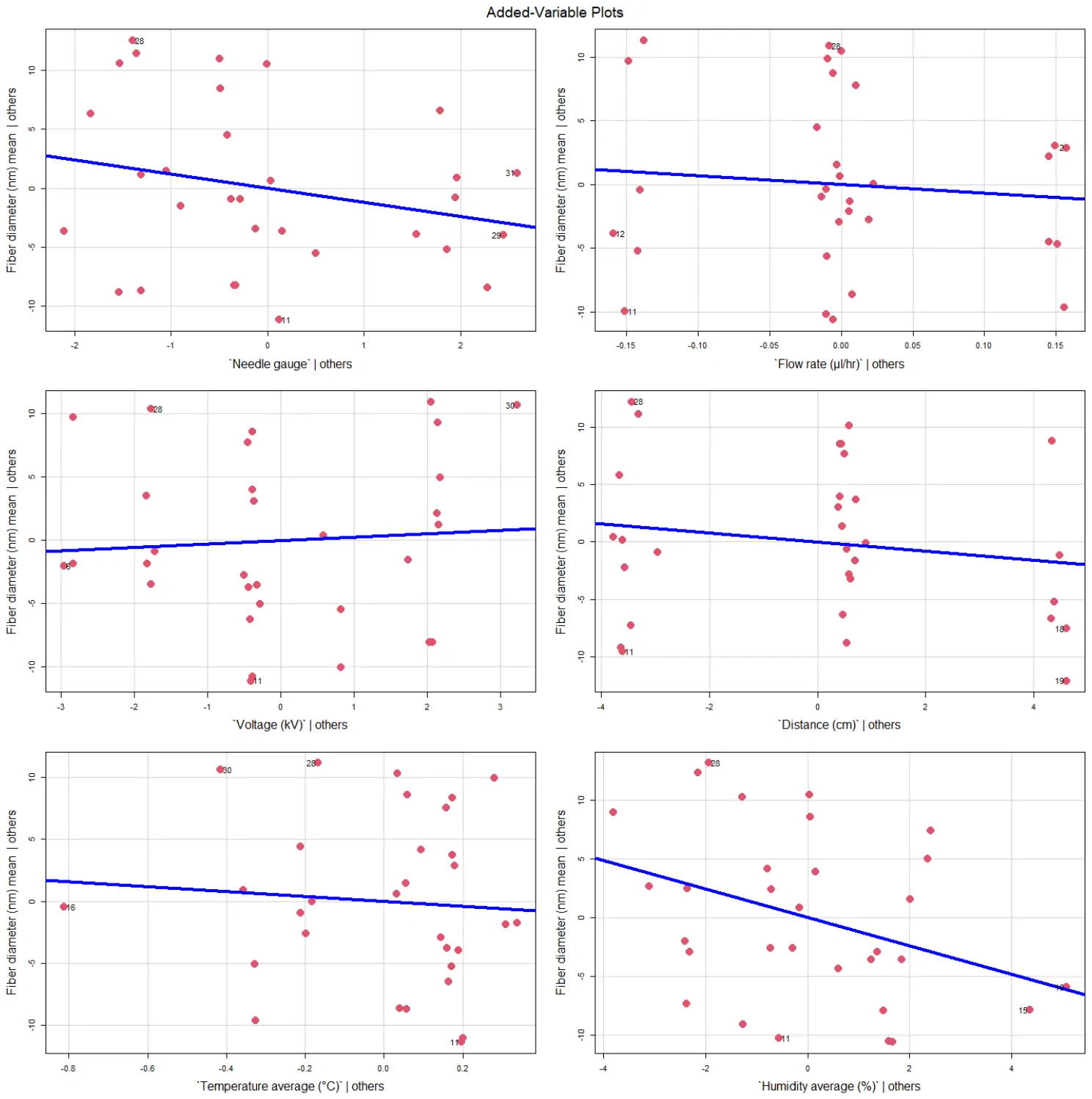
Added variable (partial regression) plots from the multiple linear regression model (n = 31) of mean fiber diameter using needle gauge, flow rate, voltage, distance as input parameters and temperature and humidity as measured additional environmental parameters. Blue lines indicate the fitted trend lines of the parameter of interest after controlling for all other parameters. Red dots: individual data.

**Table 1:** Experimental factors and levels used in the first Box–Behnken design (BBD-I) and the second Box–Behnken design (BBD-II), including needle size.

Coded Variables	Input Parameters	−1	Levels 0	1
V	Applied Voltage (kV)	15	17.5	20
Q	Flow rate (µL/h)	0.2	0.35	0.5
D	Spinning distance (cm)	8	12	16
G	Needle size (G)	20	18	16

**Table 2:** Qualitative rating scale used to assess Taylor cone shape and stability during electrospinning.

Taylor Cone (TC) Rating	Score
Very Unstable/No TC	1
Unstable/Incomplete TC	2
Borderline/Oscillating TC	3
Very Stable/Ideal TC	4

**Table 3:** Box–Behnken design matrix (BBD-I) and corresponding mean fiber diameter and standard deviation (uniformity) (n = 3).

Run Order	V	Levels Q	D	Mean Fiber Diameter (nm) ± SD (nm)
1	−1	−1	0	207.2 ± 26.2
2	−1	1	0	211.1 ± 17.6
3	1	−1	0	218.1 ± 29.1
4	1	1	0	198.9 ± 3.6
5	0	0	0	214.5 ± 14.1
6	−1	0	−1	207.5 ± 17.4
7	−1	0	1	217.9 ± 14.8
8	1	0	−1	200.9 ± 13.7
9	1	0	1	209.4 ± 3.9
10	0	0	0	204.8 ± 24.8
11	0	−1	−1	201.3 ± 5.9
12	0	−1	1	206.6 ± 13.9
13	0	1	−1	206.0 ± 19.0
14	0	1	1	204.8 ± 5.0
15	0	0	0	201.6 ± 25.1

**Table 4:** Box–Behnken design matrix (BBD-II) and corresponding mean fiber diameter and standard deviation (n = 3).

Run Order	V	Levels Q	D	G	Mean Fiber Diameter (nm) ±SD (nm)
1	0	0	−1	−1	206.35 ± 4.42
2	0	0	−1	1	205.43 ± 7.17
3	0	0	1	−1	202.79 ± 4.46
4	0	0	1	1	205.43 ± 3.77
5	−1	0	0	−1	204.01 ± 9.28
6	−1	0	0	1	216.19 ± 7.96
7	1	0	0	−1	208.89 ± 0.80
8	1	0	0	1	223.87 ± 9.95
9	0	−1	0	−1	215.17 ± 7.17
10	0	−1	0	1	205.71 ± 12.97
11	0	1	0	−1	209.45 ± 8.20
12	0	1	0	1	216.33 ± 6.80
13	−1	0	−1	1	228.40 ± 14.23
14	−1	0	−1	−1	209.54 ± 11.32
15	1	0	−1	1	228.72 ± 1.50
16	1	0	−1	−1	216.95 ± 0.00

**Table 5:** Parameters and metrics of the multiple linear regression models (n = 31) of (1) mean fiber diameter, (2) uniformity (fiber diameter variability (SD)) and (3) mean porosity using needle gauge, flow rate, voltage, distance input parameters and temperature and humidity as measured additional environmental parameters. Significance codes: ‘***’: *p* < 0.001, ‘**’: *p* < 0.01, ‘*’: *p* < 0.05, ‘.’: *p* < 0.1.

**1. Fiber diameter mean**			
**Coefficients:**	**Estimate**	**SD. Error**	***p*** **-value**
Intercept *	343.4408	124.3205	0.0108
**Needle gauge**	−1.1913	0.9846	0.2381
**Flow rate**	−6.8634	14.45	0.6391
**Voltage**	0.2706	0.7925	0.7357
**Distance**	−0.3962	0.4755	0.413
**Temperature average**	−1.9501	5.2665	0.7144
**Humidity average.**	−1.2135	0.6435	0.0715
**Residual standard error:**	7.472		
**Multiple R-squared:**	0.2537		
**Adjusted R-squared:**	0.06714		
***p*** **-value:**	0.2706		
**2. Uniformity (fiber diameter SD)**			
**Coefficients:**	**Estimate**	**SD. Error**	***p*** **-value**
**Intercept**	−128.709	85.73188	0.14632
Needle gauge *	−1.78385	0.67897	**0.01476**
**Flow rate**	−13.9207	9.96478	0.1752
Voltage **	−1.7323	0.54651	**0.00413**
**Distance**	−0.03965	0.32793	0.90477
**Temperature average**	3.25636	3.63179	0.37882
Humidity average ***	2.49568	0.44375	**8.65 × 10^−6^**
**Residual standard error:**	5.152		
**Multiple R-squared:**	0.6537		
**Adjusted R-squared:**	0.5672		
***p*** **-value:**	**0.000125**		
**3. Porosity**			
**Coefficients:**	**Estimate**	**SD. Error**	***p*** **-value**
**Intercept**	36.4964	31.024	0.251
**Needle gauge**	−0.2187	0.2457	0.382
**Flow rate**	−0.0561	3.606	0.988
**Voltage**	0.1062	0.1978	0.596
**Distance**	0.1328	0.1187	0.274
**Temperature average**	0.6536	1.3142	0.623
**Humidity average**	−0.0531	0.1606	0.744
**Residual standard error:**	1.865		
**Multiple R-squared:**	0.121		
**Adjusted R-squared:**	−0.09879		
***p*** **-value:**	0.7647		

**Table 6:** Thermogravimetric parameters of PVA nanofibers fabricated under BBD-I conditions. Values are presented as mean ± SD (n = 2–3 after exclusion of doubtful values).

Sample	T_onset_ (°C)	T_max1_ (°C)	T_max2_ (°C)
1	232.1 ± 13.7	271.3 ± 1.4	462.9 ± 2.3
2	250.0 ± 3.0	272.4 ± 4.0	463.1 ± 3.6
3	229.3 ± 7.2	273.1 ± 2.8	466.7 ± 5.9
4	248.6 ± 5.6	270.7 ± 3.2	463.7 ± 3.4
5	280.0 ± 4.5	283.6 ± 12.2	438.2 ± 2.3
6	245.5 ± 6.6	269.9 ± 3.0	464.6 ± 0.1
7	252.1 ± 4.7	261.0 ± 13.7	441.9 ± 2.1
8	246.1 ± 9.0	273.2 ± 6.2	461.0 ± 1.3
9	252.1 ± 8.7	272.2 ± 1.7	446.0 ± 11.2
10	248.5 ± 4.1	274.1 ± 2.1	451.3 ± 13.4
11	234.7 ± 5.5	269.5 ± 2.1	467.3 ± 5.3
12	245.2 ± 3.3	271.9 ± 4.0	463.3 ± 3.0
13	245.6 ± 1.8	270.7 ± 0.6	459.1 ± 10.6
14	256.3 ± 6.9	270.0 ± 1.9	451.1 ± 9.0
15	244.5 ± 6.9	271.6 ± 2.0	462.6 ± 0.1

**Table 7:** Thermogravimetric parameters of PVA nanofibers fabricated under BBD-II conditions. Values are presented as mean ± SD (n = 2–3 after exclusion of doubtful values).

Sample	T_onset_ (°C)	T_max1_ (°C)	T_max2_ (°C)
1	234.2 ± 9.1	271.3 ± 9.4	450.2 ± 7.3
2	229.0 ± 7.7	275.3 ± 9.1	462.6 ± 4.8
3	236.3 ± 8.0	270.8 ± 4.9	452.4 ± 5.9
4	242.8 ± 14.9	270.3 ± 4.9	455.1 ± 4.4
5	230.1 ± 6.7	265.7 ± 3.1	462.3 ± 7.6
6	240.0 ± 6.4	269.2 ± 1.8	456.7 ± 5.1
7	248.7 ± 3.3	274.0 ± 2.3	458.4 ± 3.3
8	228.4 ± 10.6	271.9 ± 13.1	456.0 ± 4.5
9	232.7 ± 5.9	268.7 ± 0.4	453.8 ± 3.9
10	233.2 ± 6.3	264.9 ± 4.0	435.7 ± 10.7
11	228.2 ± 12.2	259.3 ± 11.2	438.0 ± 14.4
12	231.8 ± 11.2	259.3 ± 11.2	438.0 ± 14.4
13	238.6 ± 9.1	267.0 ± 4.5	464.6 ± 2.3
14	234.7 ± 1.0	268.9 ± 1.6	462.4 ± 4.6
15	225.3 ± 5.1	294.2 ± 33.7	435.0 ± 32.0
16	228.0 ± 7.2	265.4 ± 8.1	459.9 ± 6.7

**Table 8:** DSC thermal parameters of electrospun PVA nanofiber mats prepared according to the BBD-I design. Values are presented as mean ± SD (n = 3), unless otherwise indicated.

Sample	Tg (°C)	Tp1 (°C)	ΔH1 (J/g)	Tp2 (°C)	ΔH2 (J/g)	Tp3 (°C)	ΔH3 (J/g)
1	69.71 ± 0.46	55.65 ± 5.77	4.33 ± 3.13	74.99 †	3.29 †	191.92 ± 0.25	0.97 ± 0.40
2	68.98 ± 0.58	52.24 ± 3.76	3.03 ± 0.34	81.72 †	16.73 †	192.00 ± 0.23	2.04 ± 2.41
3	70.22 ± 0.33	52.38 ± 7.31	4.45 ± 3.50	79.92 ± 0.90	8.96 ± 0.30	192.11 ± 1.08	2.59 ± 1.82
4	69.86 ± 1.30	55.76 ± 6.20	6.96 ± 5.67	73.64 †	1.64 †	192.34 ± 0.53	0.96 ± 0.80
5	69.30 ± 1.34	57.04 ± 6.18	3.60 ± 1.45	73.86 †	0.65 †	192.39 ± 0.18	0.43 ± 0.04
6	70.09 ± 0.29	59.37 ± 10.95	7.21 ± 5.55	–	–	191.75 †	2.45 †
7	68.99 ± 0.48	60.47 ± 13.84	2.62 ± 0.46	90.38 †	18.46 †	192.18 ± 1.82	1.86 ± 2.45
8	70.04 ± 1.01	56.12 ± 13.14	3.20 ± 2.11	111.97 †	0.42 †	191.19 ± 0.03	5.51 ± 4.73
9	69.58 ± 0.56	54.64 ± 3.69	2.08 ± 2.56	75.80 †	1.26 †	191.59 ± 0.45	0.61 ± 0.05
10	69.54 ± 0.85	59.88 ± 9.91	2.50 ± 1.63	75.39 †	1.60 †	187.62 ± 7.08	0.73 ± 0.66
11	70.03 ± 0.69	59.68 ± 9.69	5.53 ± 3.70	76.25 †	3.27 †	191.91 ± 1.11	1.00 ± 1.02
12	69.12 ± 0.91	52.84 ± 1.97	1.36 ± 1.18	80.30 †	1.20 †	192.52 ± 1.78	0.24 ± 0.16
13	69.90 ± 0.82	59.30 ± 11.22	3.78 ± 2.01	76.69 †	3.81 †	192.23 ± 0.83	0.58 ± 0.54
14	70.08 ± 0.55	57.99 ± 5.80	1.60 ± 2.62	75.81 †	0.62 †	192.20 ± 1.26	0.28 ± 0.21
15	68.91 ± 0.46	57.09 ± 5.97	3.57 ± 2.37	76.40 †	1.99 †	192.47 ± 0.68	0.29 ± 0.32

† Additional transition observed in only one replicate and therefore reported descriptively.

**Table 9:** DSC thermal parameters of electrospun PVA nanofiber mats prepared according to the BBD-II design. Values are presented as mean ± SD (n = 3), unless otherwise indicated.

Sample	Tg1 (°C)	Tg2 (°C)	Tp1 (°C)	ΔH1 (J/g)	Tp2 (°C)	ΔH2 (J/g)	Tp3 (°C)
1	63.27 ± 0.59	69.45 ± 0.49	55.20 ± 8.11	7.99 ± 7.86	83.09 †	15.77 †	191.96 ± 0.81
2	62.51 ± 0.57	69.24 ± 0.47	49.65 ± 3.71	4.92 ± 6.25	79.19 ± 1.63	4.68 ± 2.35	191.86 ± 0.04
3	61.95 ± 3.35	69.78 ± 1.28	54.48 ± 1.57	3.34 ± 0.88	–	–	191.47 ± 0.39
4	60.65 ± 2.80	68.47 ± 1.96	60.19 ± 1.50	4.94 ± 3.31	–	–	191.65 ± 0.25
5	63.62 ± 1.19	68.87 ± 0.86	60.46 *	4.64 *	77.96 †	14.09 †	191.78 ± 0.95
6	62.50 ± 0.51	69.30 ± 0.87	60.37 ± 7.04	9.68 ± 5.59	76.72 †	16.11 †	192.19 ± 0.27
7	64.25 *	70.23 *	61.20 *	6.32 *	–	–	192.27 *
8	62.94 ± 0.41	69.22 ± 1.70	58.33 ± 0.85	10.39 ± 3.40	–	–	191.85 ± 0.58
9	61.95 ± 3.65	68.84 ± 1.42	54.30 ± 6.02	8.15 ± 13.01	87.56 †	24.48 †	191.04 ± 0.73
10	62.89 ± 0.97	69.75 ± 0.34	60.50 ± 11.34	11.31 ± 9.19	72.52 ± 1.30	21.10 ± 4.75	192.37 ± 0.30
11	63.61 ± 4.49	69.34 ± 2.89	58.68 ± 1.65	4.09 ± 2.09	–	–	191.42 ± 0.37
12	64.12 ± 1.35	69.95 ± 1.46	58.05 ± 2.29	4.64 ± 1.72	–	–	190.49 ± 0.16
13	63.44 ± 0.82	69.22 ± 0.39	56.53 ± 5.77	10.41 ± 6.20	79.07 †	5.44 †	191.69 ± 0.26
14	63.32 ± 0.10	69.80 ± 0.57	57.33 ± 2.54	10.42 ± 0.86	–	–	192.14 ± 0.18
15	63.43 ± 0.05	69.01 ± 0.18	48.60 ± 2.95	1.82 ± 0.25	81.92 ± 4.13	10.14 ± 6.09	191.67 ± 0.36

* Single measurement available; the value was not included in comparative interpretation; † Additional transition observed in only one replicate and therefore reported descriptively.

## Data Availability

The raw data supporting the conclusions of this article will be made available by the authors on request.

## Supplementary Materials

The following supporting information can be downloaded at: https://www.mdpi.com/article/10.3390/polym18162018/s1, Figure S1: SEM micrographs of electrospun PVA nanofibers fabricated under BBD-I conditions labelled 1–15, illustrating variations in fiber diameter, uniformity, and defect formation; Figure S2: SEM micrographs of electrospun PVA nanofibers fabricated under BBD-II conditions, highlighting the effect of needle size on fiber morphology; Figure S3: Representative Taylor cone images and qualitative stability ratings for the 15 experimental runs of BBD-I. Figure S4: Representative Taylor cone images and qualitative stability ratings for the 16 experimental runs of BBD-II; Figure S5: Mean fiber diameter (±SD) for the 15-run Box–Behnken design (BBD-I) as a function of applied voltage, flow rate, and spinning distance (n = 3). Figure S6: Mean fiber diameter (±SD) for the 16-run Box–Behnken design (BBD-II) including needle size as an additional factor (n = 3).
