# Supplementary material for: Multiparametric Optimization of Fabrication of Electrospun PVA Nanofibers for Utilization as Wound Dressing Mats
Source: Polymers (Basel). 2026 Aug 20;18(16):2018. doi: 10.3390/polym18162018 (PMC13517352; doi:10.3390/polym18162018)
Supplement: Supplementary file 1 [file polymers-18-02018-s001.zip › polymers-4476694-supplementary.pdf]

Figure S1

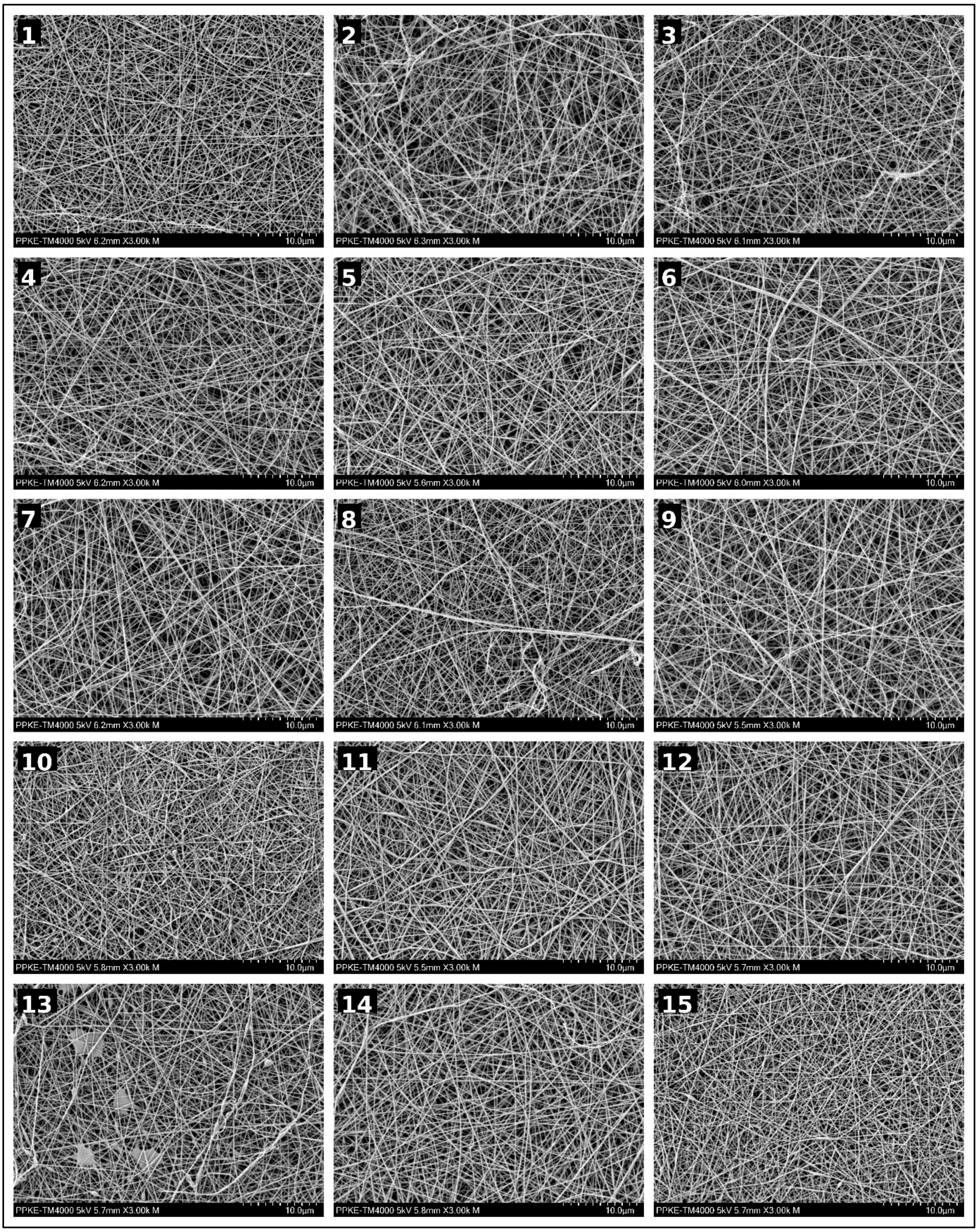

Figure S1. SEM micrographs of electrospun PVA nanofibers fabricated under BBD-I conditions labelled 1-15, illustrating variations in fiber diameter, uniformity, and defect formation.

Figure S2

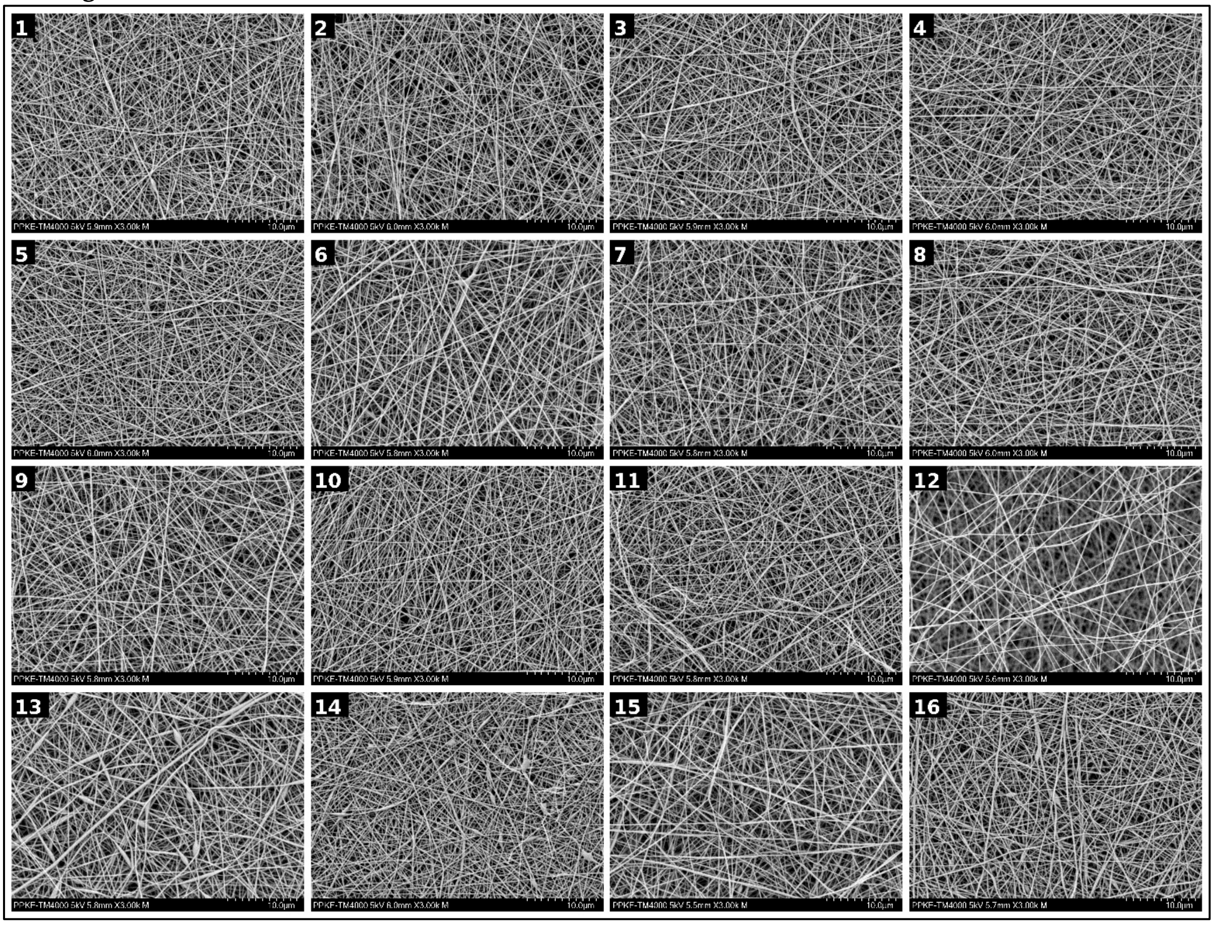

Figure S2. SEM micrographs of electrospun PVA nanofibers fabricated under BBD-II conditions, highlighting the effect of needle size on fiber morphology.

Figure S3

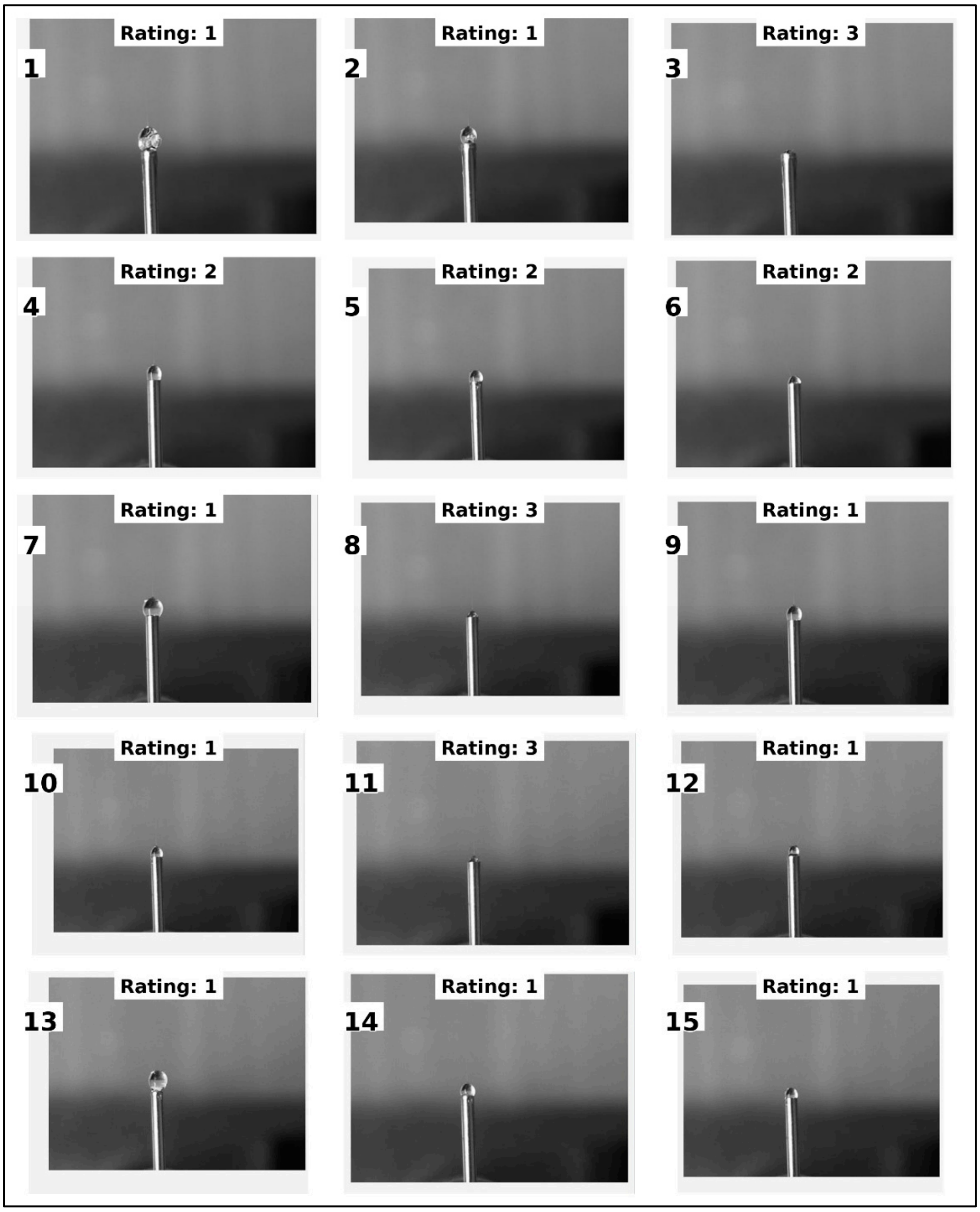

Figure S3. Representative Taylor cone images and qualitative stability ratings for the 15 experimental runs of BBD-I

Figure S4

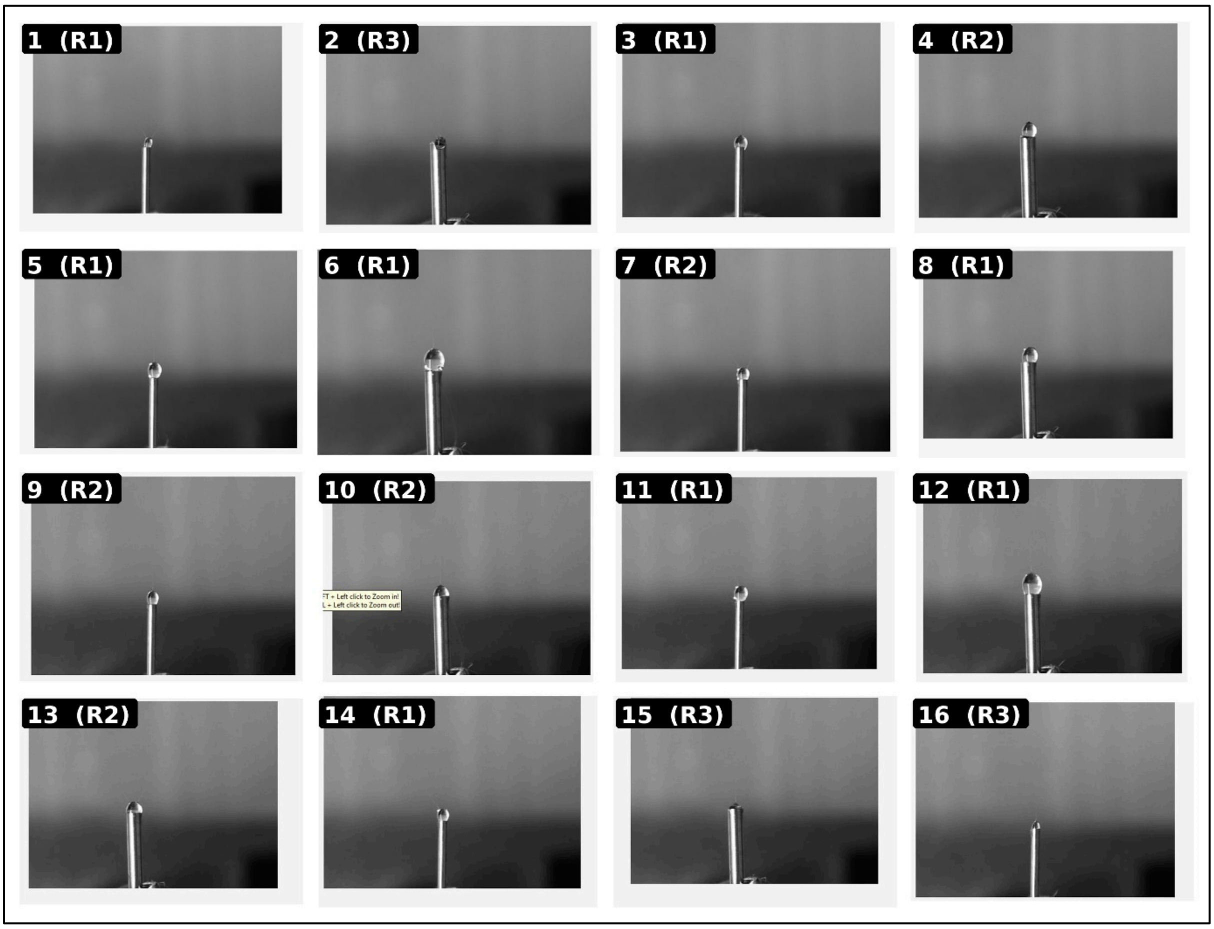

Figure S4. Representative Taylor cone images and qualitative stability ratings for the 16 experimental runs of BBD-II

Figure S5

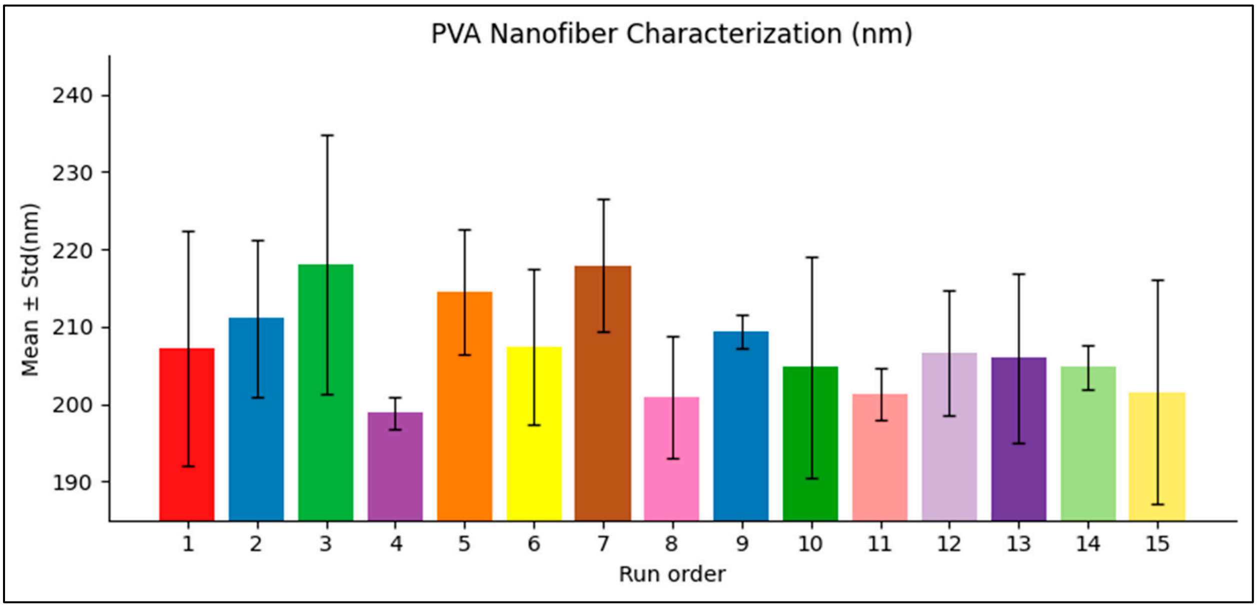

Figure S5. Mean fiber diameter (± SD) for the 15-run Box–Behnken design (BBD-I) as a function of applied voltage, flow rate, and spinning distance (n = 3).

Figure S6

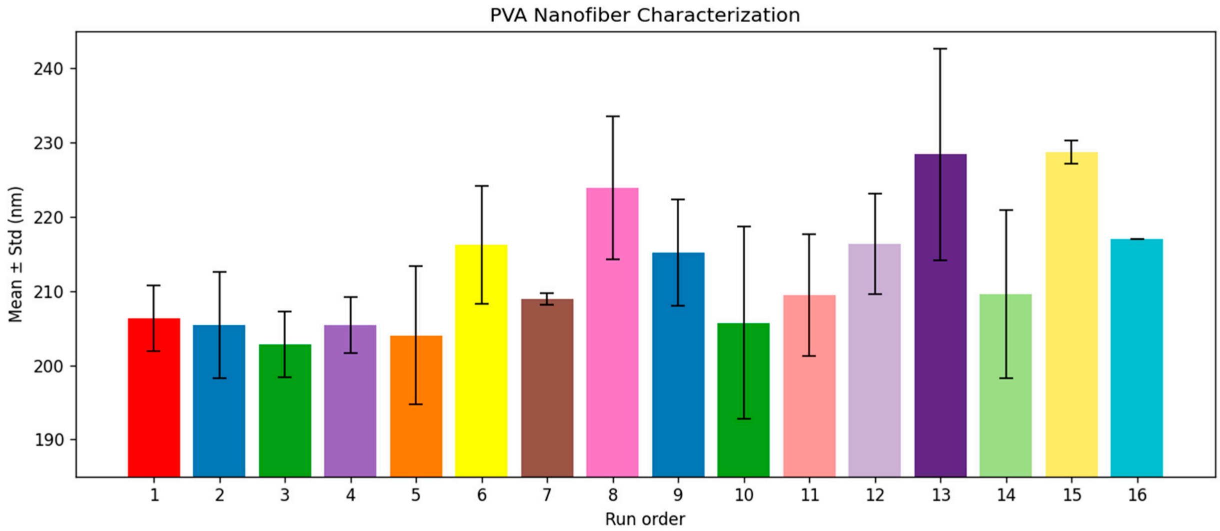

Figure S6. Mean fiber diameter (± SD) for the 16-run Box–Behnken design (BBD-II) including needle size as an additional factor (n = 3).
